# Multifunctional Hydrogel with Phytochemicals and Silver Nanoparticles for Promoting Scar-Free Wound Healing

**DOI:** 10.3390/gels12080719

**Published:** 2026-08-13

**Authors:** Devadass Jessy Mercy, Koyeli Girigoswami, Pazhani Durgadevi, Venkatakrishnan Kiran, Agnishwar Girigoswami

**Affiliations:** 1 Medical Bionanotechnology, Faculty of Allied Health Sciences, Chettinad Hospital and Research Institute, Chettinad Academy of Research and Education (CARE), Kelambakkam, Chennai 603103, Tamil Nadu, India; jessy331mercy@gmail.com (D.J.M.);; 2 Medical Bionanotechnology Lab, Department of Obstetrics and Gynecology, Saveetha Medical College and Hospital, Saveetha Institute of Medical and Technical Sciences, Thandalam, Chennai 602105, Tamil Nadu, India; koyelig@gmail.com

**Keywords:** wound healing, antioxidant, antimicrobial, angiogenesis, health care, tissue regeneration

## Abstract

Background/objectives: Delayed wound healing, along with excessive scar formation, is the major clinical drawback due to the presence of stubborn bacteria, oxidative stress, prolonged inflammation, and irregular tissue regeneration. Utilizing nanomaterial-based wound dressings offers significant advancements and minimal cytotoxicity, but also presents issues such as poor biocompatibility, low solubility, and reduced permeability. These factors limit the effectiveness of nanomaterial-based wound dressings in promoting complete tissue regeneration. To overcome these limitations, plant-derived bioactives are integrated with nanomaterials within a hydrogel cage to enhance antibacterial activity, mitigate oxidative stress and inflammation, and promote tissue regeneration. Methods: A multifunctional alginate–gelatin hydrogel incorporating silver nanoparticles and plant extracts (AG-AgNP-PE) was developed to promote scar-free wound healing, alongside a plant extract-free silver nanoparticles-loaded hydrogel for comparative evaluation of the functional contribution of plant bioactives. The biological performance of the formulated hydrogels was systematically evaluated through antioxidant, antimicrobial, and antibiofilm assays, while in vitro cytocompatibility and proregenerative activity were assessed using MTT and Alamar Blue assays, live/dead cell imaging, and a scratch-wound assay, complemented by in vivo evaluation in zebrafish embryos. Results: Pro-angiogenic activity was further investigated using the CAM model, and therapeutic efficacy was validated in an in vivo rat burn wound model through microscopic wound assessment, histopathological examination, and biochemical assays. Conclusions: Among the hydrogels, AG-AgNP-PE exhibited superior performance across all key properties, highlighting the synergistic effect of the nanomaterial combined with plant extracts within hydrogel cages and positioning it as a promising multifunctional wound dressing for rapid tissue regeneration and scar-free wound healing, suitable for advanced wound management.

## 1. Introduction

Burn wound management is a crucial challenge in wound healing. These wounds are limited by a critical pathological environment, such as prolonged inflammation and persistent infections [1,2]. These wounds affect the body parts both locally and systemically, transforming into critical clinical challenges. The localized injury often damages the blood vessels and cells that are essential in producing the blood supply. This environment complicates the healing process by trapping pro-inflammatory macrophages, making it difficult for the tissues to undergo regeneration [3]. The progress of healing and tissue repair specifically depends on external and internal factors such as age, hormonal fluctuations, nutritional status, infections, prolonged inflammation, oxidative stress, and bacterial formation, which ultimately leads to tissue scarring [4]. Based on these factors, burn wounds are classified into five different types, depending on their depth of cause and damage [5]. Despite the advancements noted in medical sciences, numerous limitations still affect and delay the healing period of wounds, even causing mental and physical trauma. Hence, early determination of the severity of the injury is essential to provide immediate treatment that promotes early wound recovery. Systemic injury primarily affects the processes of inflammation, immune response, metabolism, infection, and mental health, which further alter the function of organs and can eventually lead to mortality [6,7]. Scar-free wound healing can open up the opportunity for burn victims to lead their lives with high dignity, because burn scars create social isolation and psychological setbacks in such affected individuals [8].

The nanoform of silver nanoparticles (AgNPs) is a well-known antimicrobial agent that can be incorporated into polymeric hydrogels to combine their potent antimicrobial properties with the biocompatible, hydrated, and wound-supportive properties of hydrogel matrices [9,10]. Due to their antimicrobial qualities, controlled release mechanism, and ability to stimulate tissue regeneration, hydrogels developed with nanomaterials have become a potential wound treatment. Although their clinical translation is still difficult, recent developments have also resulted in the development of hybrid and smart wound dressings [11]. The polymeric hydrogel network provides nanoscale voids that act as nucleation and growth sites for AgNPs, stabilizing the nanoparticles and potentially reducing aggregation while enabling their controlled retention and release profile [12]. In biological environments, AgNPs exert an antimicrobial effect primarily through the gradual release and oxidation of silver ions, which penetrate microbial cells, disrupt membrane potentials, and inhibit essential cellular processes, thereby inhibiting cell growth and further division. Hydrophilic hydrogel matrices made of natural polymers maintain a moist environment conducive to tissue regeneration during wound-healing applications while facilitating sustained availability of AgNPs at the wound site [13,14]. Thus, encapsulation of AgNPs within hydrogel matrices aims to achieve localized and prolonged antimicrobial activities to improve the stability, delivery, and release of AgNPs, limiting uncontrolled exposure and simultaneously providing a biocompatible scaffold conducive to wound healing.

To improve a partial-thickness burn wound, a silver sulfadiazine (SSD)-loaded nanosuspension was incorporated into a chitosan hydrogel by Barkat et al. [15]. Although SSD was the standard drug used for burn wound management, it often had limitations, including low skin penetration, skin irritation, and poor water solubility. In order to overcome the barrier, the researchers optimized the size of the nanoparticles, which were then loaded into the chitosan nanogel, resulting in better drug release, distribution, and easy extrusion, specifically for topical application. This proves that the combined nanotechnological application drastically improved the therapeutic performance of SSD, ultimately increasing the drug efficacy and treatment efficiency, leading to alternative clinical management of partial-thickness burn wounds [15]. Abdullah et al. developed a glutathione-acacia alginate nanogel to enhance the healing of acute wounds by reducing oxidative stress, a crucial factor that alters and delays tissue repair and regeneration [16]. Glutathione has superior antioxidant properties; however, it is poorly stable, has low penetrability, and is further incorporated into alginate nanogel. The hydrogel was observed to be smaller in size, provided high swelling efficiency, and increased stability over 24 h, ensuring sustained antioxidant activity. The hydrogel was further tested in an animal model, where it promoted wound closure, as reflected in the key healing biomarkers such as VEGF, TGF-β1, and collagen. It also promoted organized inflammatory responses, further acting as a promising therapeutic material for acute wound management [16]. Similarly, a silver-coated gelatin/fucoidan nanogel was developed to prevent bacterial contamination as well as to promote tissue regeneration [17]. The developed formulation effectively inhibited bacterial growth, such as *Escherichia coli* and *Staphylococcus aureus*, and promoted the proliferation and migration of L929 fibroblast cells, reflecting the process of tissue regeneration. The nanoformulation acted as an effective and safe dressing material for wound management [17].

Although the nanomaterials possess superior antimicrobial activity, enable the sustained release of drugs, and can even initiate the ability to promote tissue formation, their clinical application is limited by dose-dependent cytotoxicity [18], instability [19], oxidative stress, infections [20], and insufficient production of critical biological and wound-healing pathways [21]. Plant extracts contain active biocompounds that modulate biological activity and promote wound-healing pathways, exhibiting crucial responses like anti-inflammatory, antioxidant, and antimicrobial properties, along with increased angiogenic activity, enhanced collagen deposition, and fibroblast proliferation [22,23]. They produce synergistic effects when combined with nanomaterials, resulting in improved stability (13) and release of phytochemicals In addition, plant extracts reduce limitations such as toxicity and oxidative stress [24]. This synergistic effect promotes rapid closure of wounds, superior tissue formation, improved vascularization, enhanced extracellular matrix, increased remodeling, reduced inflammation, and reduced scarring compared with nanomaterials alone [25]. Recent advances in multifunctional wound dressings have shown that combining various therapeutic techniques on a single platform can effectively address the complicated pathophysiology of chronic wounds. A recently developed thermoelectric bio-dressing utilized photothermal antibacterial therapy, self-powered electrical stimulation, and antioxidant-mediated immunoregulation to eliminate bacterial infection while also reducing oxidative stress, regulating inflammation and stimulating vascular growth. Although these advanced systems provide superior therapeutic effects, they require complex device manufacture, external NIR activation, and thermoelectric components, which may affect cost and restrict manufacturing ability. In order to provide antimicrobial, antioxidant, antibiofilm, pro-angiogenic, and tissue regenerative functions without the need for an external power device, Yuan et al. developed a simple alginate–gelatin biomaterial-based dressing material by incorporating AgNPs along with plant extracts [26]. Because of their superior biocompatibility, biodegradability, moisture retention, and exudate absorption [27], conventional alginate–gelatin hydrogels are well known for being appropriate scaffolds for wound healing. In order to improve therapeutic efficacy [28], these hydrogels frequently need to be supplemented with additional bioactive chemicals because of their weak antibacterial and antioxidant capacities [29]. Based on recent research, alginate-based nanoparticles, along with plant extract-derived phytochemicals, further boost cell tissue regeneration, antioxidant properties, and biocompatibility. By combining AgNPs with numerous plant-derived phytochemicals, the resulting hydrogel possesses the capacity of a traditional alginate–gelatin hydrogel [30].

Hence, in this study, we developed alginate and gelatin hydrogels incorporated with silver nanoparticles and bioactive plant extracts, including the *Calendula officinalis* flower, plantain peel, aloe vera, and curcumin, with the aim of promoting scar-free wound healing. Six different hydrogel formulations were designed, ranging from the basic alginate, gelatin, and silver nanoparticle hydrogel to formulations containing individual and combined plant extracts. This strategy was developed to evaluate whether the synergistic effect of multiple phytochemicals enhances scar-free wound healing, promotes superior angiogenesis, reduces inflammation, and improves tissue remodeling compared to the nanomaterial-based hydrogel alone. Comparisons of these nanoformulations highlight the importance of plant bioactive compounds in overcoming the limitations of nanomaterials and promoting scar-free tissue regeneration. The compositions of the formulations are varied to offer the best possible combinations for burn-wound treatment and abbreviated as follows: AG-AgNP: (alginate + gelatin + silver nanoparticle), AG-AgNP-PE: (alginate + gelatin + silver nanoparticle + combined plant extracts), AG-AgNP-FL: (alginate + gelatin + silver nanoparticle + flower extract), AG-AgNP-PL: (alginate + gelatin + silver nanoparticle + plantain peel extract), AG-AgNP-AL: (alginate + gelatin + silver nanoparticle + aloe vera), and AG-AgNP-CU: (alginate+ gelatin+ silver nanoparticle + curcumin).

## 2. Results and Discussion

### 2.1. Preparation of the Hydrogels

The hydrogels AG-AgNP, AG-AgNP-PE, AG-AgNP-FL, AG-AgNP-PL, AG-AgNP-AL, and AG-AgNP-CU were prepared and used for further experiments.

### 2.2. Physicochemical Characterization of the Hydrogels

**Figure 1:**
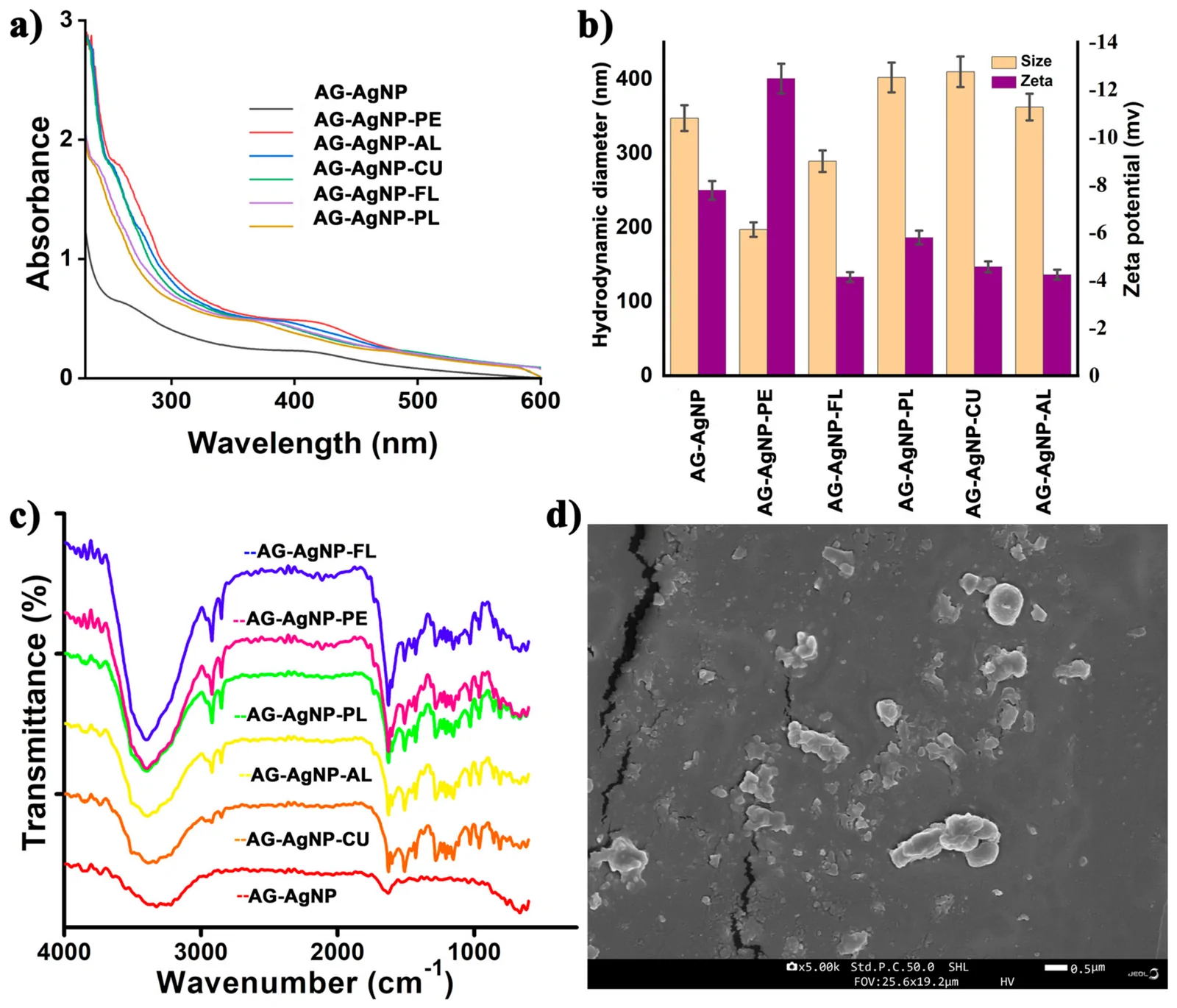
Primary characterization of the synthesized hydrogels. (**a**) Schematic representation of the absorption spectra of all the hydrogels; (**b**) hydrodynamic diameter and zeta potential obtained for the hydrogels; (**c**) FTIR spectra obtained for all hydrogels; and (**d**) scanning electron micrograph of hydrogel AG-AgNP-PE (scale 0.5 µM).

The prepared hydrogels were characterized to evaluate their optical physicochemical properties using UV-visible spectroscopy, particle size analysis, zeta potential measurements, and FTIR analysis to identify the functional groups and their chemical bonds. The absorption spectra showed a prominent broad absorption between 405 and 450 nm, peaking at 416 nm (Figure 1a) for all the synthesized hydrogel formulations, confirming the successful blending of silver nanoparticles in hydrogels. At the same time, hydrogels containing plant extracts in the form of AG-AgNP-PE, AG-AgNP-PE, AG-AgNP-FL, AG-AgNP-PL, AG-AgNP-AL, and AG-AgNP-CU showed a broad peak in the 200–330 nm range (Figure 1a), indicating the presence of bioactive phytochemicals such as phenolic compounds and flavonoids. Overall, the UV-visible absorption spectra indicate the presence and proper blending of silver and plant extracts in the hydrogel, and these findings are consistent with previous reports [31,32].

The colloidal properties and surface charges of the hydrogels are graphically represented in Figure 1b. The hydrodynamic diameter of the hydrogel AG-AgNP-PE was recorded as 187 nm, whereas AG-AgNP showed a particle size of 347 nm. The remaining hydrogels exhibited sizes ranging from 280 to 410 nm. Interestingly, the smaller hydrodynamic size of AG-AgNP-PE can be attributed to the synergistic effect of the plant extracts in the formulation. It was widely reported that plant extracts contain polyphenols, flavonoids, and various organic acids that can participate in crosslinking and cause a reduction in colloidal size [33,34]. These findings also indicate a reduction in particle size mediated by phytochemicals, acting as a natural reducing agent, resulting in an enhanced distribution of nanoparticles with their colloidal stability [35]. The zeta potentials were measured to assess the surface charge, and the results are plotted in Figure 1b. The formulated AG-AgNP-PE shows a zeta potential of −12.47 mV, indicating a mildly stable surface charge of the hydrogel after stabilizing the encapsulated AgNPs along with the plant extracts. The transmittance bands corresponding to specific functional groups are examined using FTIR/ATR spectroscopy (Figure 1c) and are presented in Table 1 to confirm the synthesis. Mark A corresponds to the presence of functional groups in all formulated hydrogels, including AG-AgNP, whereas section B corresponds to the transmittance bands assigned to chemical bonds, and functional groups are present in hydrogels containing plant extracts. The functional groups observed in the AG-AgNP hydrogel indicate the presence of a polymeric matrix and crosslinking [36]. The presence of phytochemicals and their involvement in hydrogel formation were also reflected in the FTIR/ATR bands (Table 1). Figure 1d depicts an SEM image of AG-AgNP-PE, illustrating that the crosslinked hydrogel with a porous structure encapsulates the silver nanoparticles.

### 2.3. Release Profile

**Figure 2:**
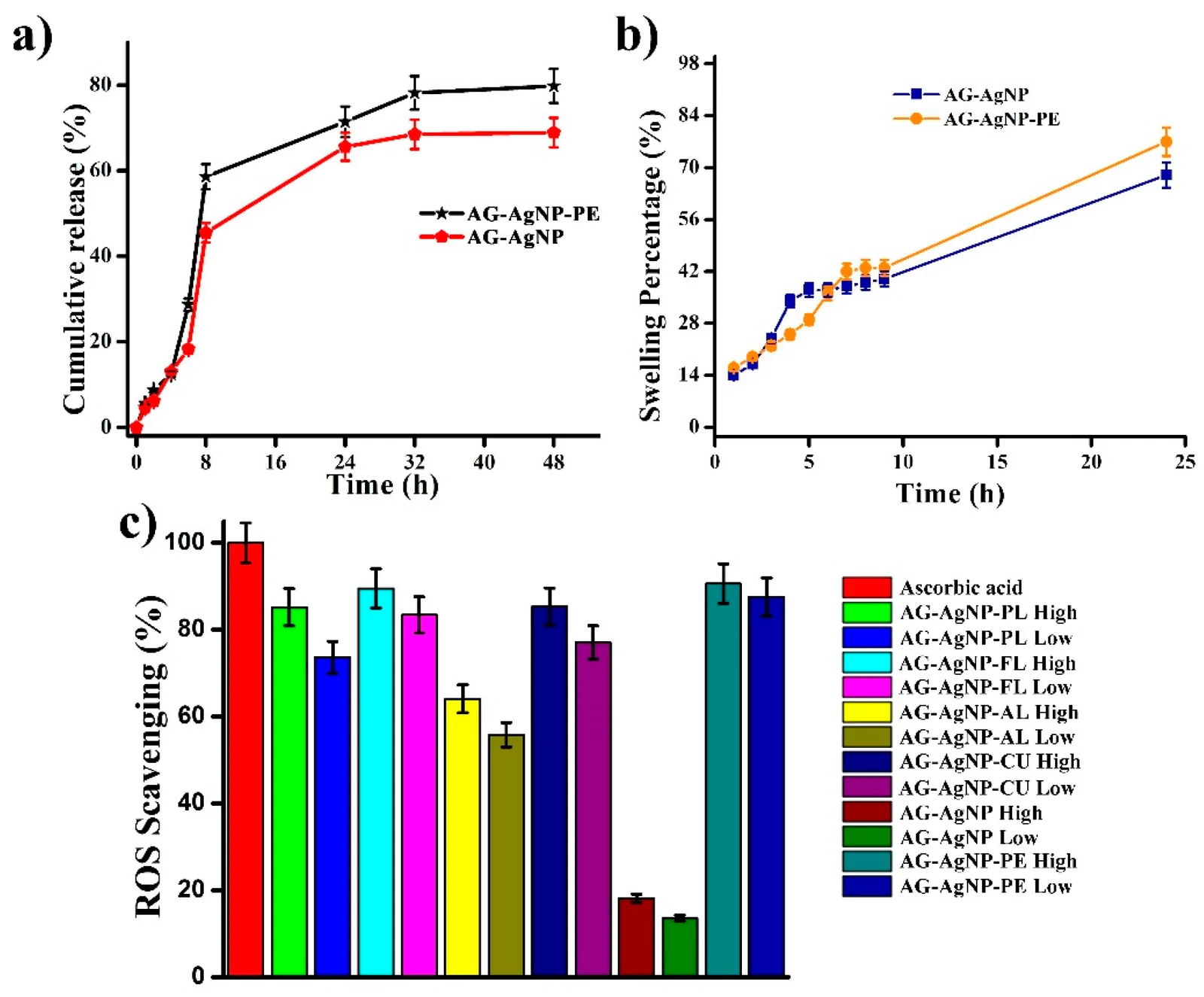
Represents the overview of the hydrogel’s performance. (**a**) Represents the cumulative percentage of drug release studied for 48 h, (**b**) represents the percentage of swelling capacity of the hydrogels, and (**c**) illustrates the percentage of ROS scavenging activity of the extracts and the prepared hydrogels.

The cumulative release profile of AgNPs encapsulated in the hydrogels AG-AgNP and AG-AgNP-PE was analyzed for 48 h. The formulated hydrogels showed an initial burst release up to 8 h, followed by slower release from the entrapped silver nanoparticles, and later became a steady state after 32 h, exhibiting a prolonged and sustained release profile, as represented in Figure 2a. The hydrogel AG-AgNP-PE showed a cumulative release of 80%, whereas AG-AgNP showed a cumulative release of 70%. The increased release observed in AG-AgNP-PE indicates that the alteration in the hydrogel matrix was enhanced due to the presence of plant extracts. These findings prove that both hydrogels have the capacity to provide prolonged release, as typical therapeutic delivery systems do. In addition, the hydrogel AG-AgNP-PE exhibited superior sustained release, making it a highly promising material for wound-healing applications.

### 2.4. Swelling Behavior

The swelling of the hydrogels AG-AgNP and AG-AgNP-PE was measured for a 24 h period. The hydrogels showed time-dependent fluid uptake but with different swelling patterns, where AG-AgNP exhibited rapid swelling in the initial period, followed by a gradual decrease, with a maximum swelling percentage of 63% at 24 h. However, the hydrogel AG-AgNP-PE revealed a gradual and sustained fluid uptake throughout the study, reaching a maximum swelling percentage of 80% at 24 h, which is represented in Figure 2b. These findings indicate that the hydrophilic nature and structural alterations, initiated by the incorporated plant extracts, enhanced the swelling ability of the hydrogel. This proves that the hydrogel AG-AgNP-PE promotes wound healing by providing the essential moisture in the wound environment. These findings were consistent with the previously reported studies, indicating that plant extracts infused into hydrogel exhibit a higher swelling ratio, indicating the dual role of promoting the sustained release of bioactive materials as well as inhibiting pathogens and creating a moisture environment [37].

### 2.5. Antioxidant Activity by DPPH Assay

The DPPH assay was used to evaluate the antioxidant activity of the plant extracts, such as plantain peel, aloe vera, *calendula*, curcumin, and the prepared hydrogels. Antioxidants play a crucial role in neutralizing free radicals by donating electrons or hydrogen atoms. The phytochemicals and other antioxidant compounds present in the plant extracts promote healing by controlling and maintaining the level of oxidative stress production. This decreases the inflammatory phase, increases the proliferation of fibroblast cells, promotes angiogenesis and collagen synthesis, and accelerates tissue regeneration. Ascorbic acid was used as a positive control and exhibited 95% scavenging activity. Among the hydrogels, AG-AgNP-PE showed the highest antioxidant activity, with 90% and 87% ROS scavenging activity at its higher and lower concentrations, respectively, which was similar to the positive control. Similarly, the plant extracts like calendula, plantain peel, curcumin, and aloe vera exhibited scavenging activities of 89%, 85%, 85%, and 64% at higher concentrations, whereas the hydrogel AG-AgNP showed a comparatively lower scavenging activity of 18% and 13% at higher and lower concentrations, respectively. The % of ROS scavenging activity of the hydrogels and extracts was represented in Figure 2c. Hence, these findings prove that the enhanced antioxidant activity of the hydrogel AG-AgNP-PE was due to the combination of plant extracts present. They also confirm that AG-AgNP-PE exhibits strong antioxidant activity, which neutralizes the free radicals and reduces oxidative stress, promoting the rapid healing of wounds by increasing the production of healthy cells due to the presence of natural antioxidant compounds in the plant bioactives, as reported earlier [38].

### 2.6. Cell Viability and Wound-Healing Assay

**Figure 3:**
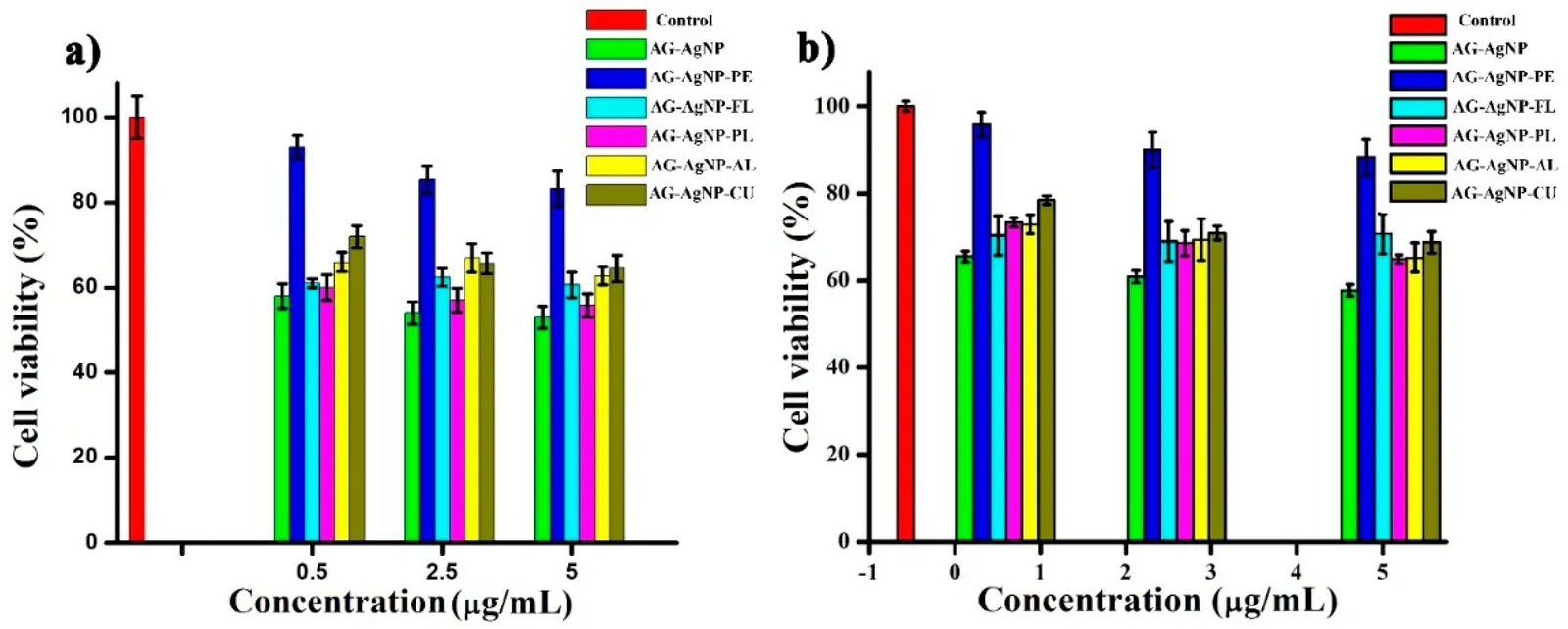
Cytotoxicity activity of the hydrogels. (**a**) Graphical representation of the hydrogels obtained by the MTT assay; (**b**) graphical representation of the Alamar Blue assay performed on the cell line.

The cell viability of the hydrogels was studied on the V79 cell line using the MTT assay, as stated earlier, and the % viability is represented in Figure 3a. Assessing the cytocompatibility of the hydrogels is crucial as it evaluates the effect on wound healing and enhances the growth of cells without causing toxic effects. Similar to antioxidant activity, cell viability is more important as the proliferation of cells is crucial for complete and rapid healing.

Among all the hydrogels tested, AG-AgNP-PE exhibited the greatest viability of 83% even at its highest concentration (5 µg/mL), indicating superior cytocompatibility compared to AG-AgNP. Similarly, the hydrogels AG-AgNP-FL, AG-AgNP-PL, AG-AgNP-AL, and AG-AgNP-CU showed greater viabilities of 61%, 60%, 66%, and 72%, respectively, at the highest concentration, which was also greater than that of AG-AgNP. These findings prove that plant extract-loaded hydrogels significantly promote tissue regeneration, as reflected in the higher cell viability observed in the combined hydrogel, supporting the fibroblast proliferation that ultimately leads to wound healing. Similar findings have been reported, stating that phyto-infused wound dressings promoted fibroblast activation, improving the viability of cells by minimizing ROS generation and promoting the essential healing environment [39].

The Alamar Blue assay is crucial for determining the biocompatibility of the nanomaterials and their regenerative capacity by observing the growth of cells in vitro. The non-fluorescent blue dye, also called resazurin, is reduced to a fluorescent pink dye, resorufin, by living cells via cytosolic and mitochondrial enzymes, reflecting metabolic activity. So, to determine the biocompatibility and cytotoxic effects of all the hydrogels, an Alamar Blue assay was performed on the cell line. The study indicates that AG-AgNP-PE showed 88% biocompatibility even at high concentration (5 µg/mL), whereas the hydrogel AG-AgNP showed only 57%. In comparison with the hydrogels, AG-AgNP-FL, AG-AgNP-PL, AG-AgNP-AL and AG-AgNP-CU showed 70%, 65%, 65%, and 68%, respectively. Meanwhile, AG-AgNP-AG showed less cell viability at all concentrations, and these findings indicate that the natural materials were not toxic; instead, they were highly biocompatible, which increased the viability of the cells, consistent with the wound healing results shown in Figure 3b. This corroborates the MTT assay and proves that the combined hydrogel is more effective in promoting rapid wound healing.

Studies have already demonstrated that the alginate-based hydrogel, along with plant bioactives, promotes significant biocompatible activity, followed by anti-mycobacterial activity, which effectively promotes healing by inhibiting the formation of pathogens [40]. Hence, it is proven that the hydrogel AG-AgNP-PE possesses the ability to promote tissue regeneration due to its biocompatible nature.

### 2.7. Wound-Healing Assay

This crucial assay monitors the capacity of the materials designed for wound repair by assessing their migration and proliferation ability and imitating the critical stage during wound healing. Treatment with two different concentrations was given once every day, and the scratch closure was monitored and photographed using an inverted microscope (Olympus CKX41) (Tokyo, Japan) at 40× magnification. Among all the developed hydrogels, the treatment with AG-AgNP-PE hydrogel promoted rapid wound closure at 72 h in both concentrations. The individual extract-loaded hydrogels, AG-AgNP-FL, AG-AgNP-PL, AG-AgNP-AL, and AG-AgNP-CU, also promoted wound closure more than the hydrogel AG-AgNP. Hence, the findings demonstrate that the AG-AgNP-PE hydrogel, which combines silver nanoparticles with various plant bioactives, has a superior capacity to enhance migration, thus promoting rapid healing. Compared with the hydrogel AG-AgNP, the synergistic effects of the plant bioactives and nanomaterials significantly improved the closure of wounds, thus supporting the enhanced regenerative potential of the formulation. Table 2 represents the percentage of wound closure observed at 72 h over the experimental period.

**Figure 4:**
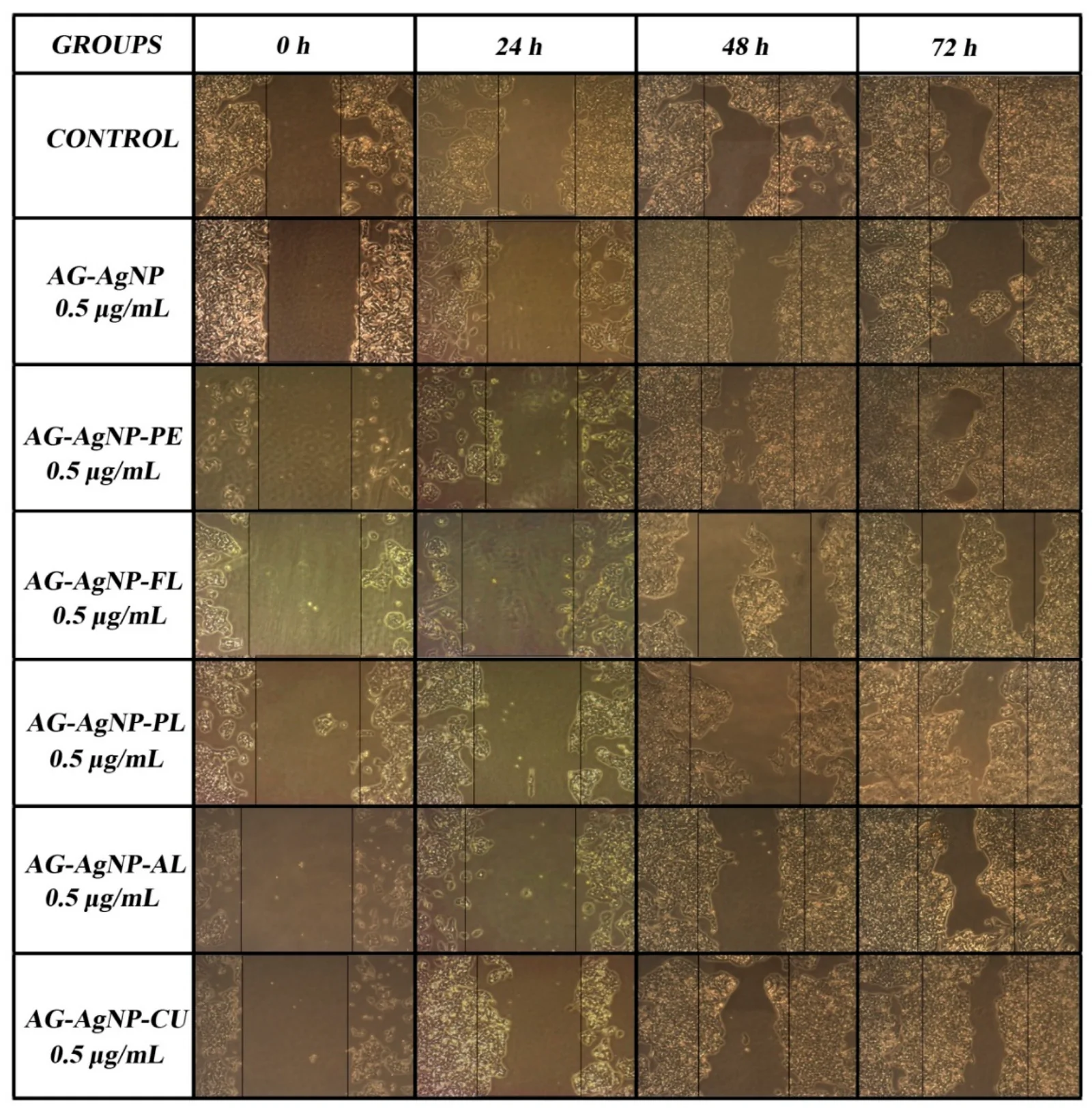
Wound healing activity promoted by the developed hydrogels with a lower concentration (0.5 µg/mL).

**Figure 5:**
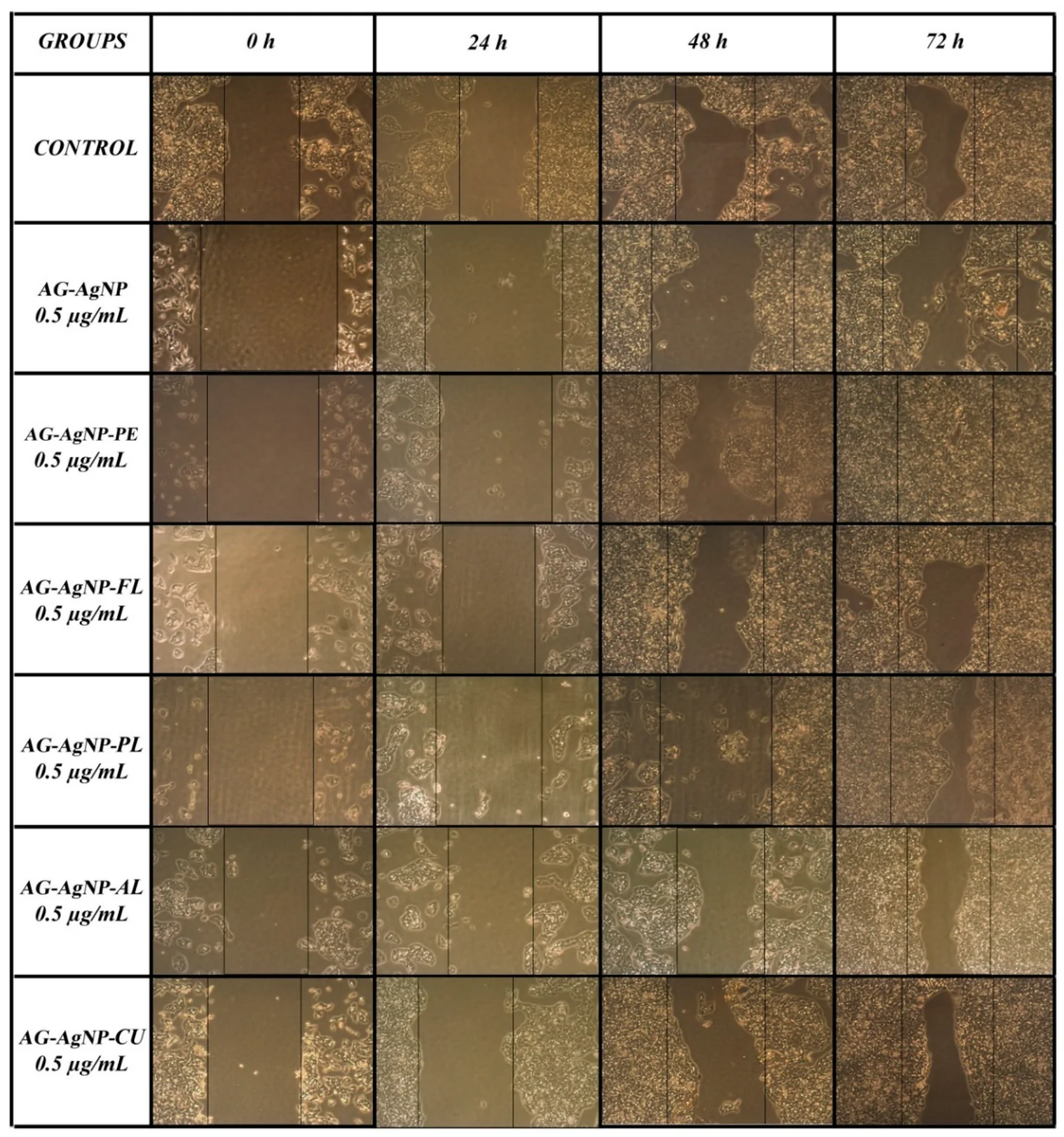
Wound healing activity promoted by the developed hydrogels with a higher concentration (5 µg/mL).

Therefore, this indicates that the hydrogel AG-AgNP-PE is highly effective and more specific in accelerating tissue regeneration, leading to rapid wound healing. Figure 4 and Figure 5 show the wound closure observed in cells treated with all the hydrogel formulations at both the lowest (0.5 µg/mL) and the highest concentrations (5 µg/mL).

The figures exhibit the variation in wound closure observed over the experimental period, underlining the wound healing potential of each hydrogel formulation at both concentrations. However, previous studies demonstrate that the plant bioactive-combined nanomaterials improve wound closure, rather than only silver-based nanomaterials [41]. The 96% and 99% wound closure at low and high doses, respectively, demonstrate that the synergistic effect plays a major role in promoting wound healing compared with the nanomaterial-incorporated silver alone.

### 2.8. Viability Assessment by Live–Dead Assay

**Figure 6:**
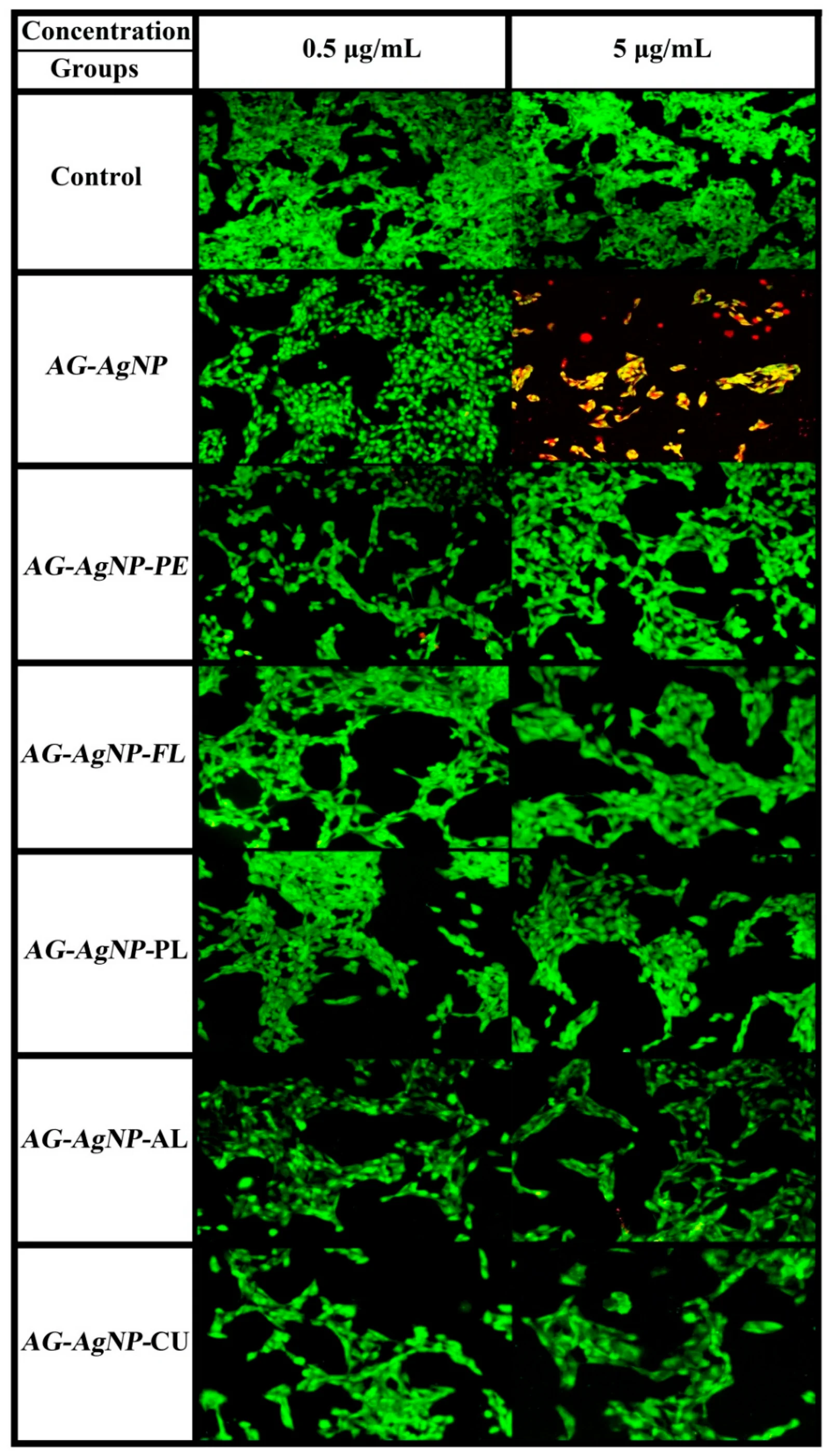
Fluorescence image panel of the live–dead assay treated with all hydrogels.

To distinguish live cells from dead cells, a cytotoxicity assay was performed to evaluate the toxic effect of the hydrogels on the cell line. This assay provides the effects of cytotoxicity and biocompatibility of the hydrogel specifically designed for wound healing. The assay was carried out by treating two different concentrations of all the hydrogels (0.5 and 5 µg/mL). Cell death was observed in the plates treated with a higher concentration of AG-AgNP, while mild morphological changes were noted in the plates treated with a lower concentration of AG-AgNP. In contrast, the other hydrogels showed no indication of cell killing at both concentrations, with AG-AgNP-PE supporting more live cells in both concentrations. These findings suggest that the selected plant extracts enhance wound healing, as the combined hydrogel promotes cell growth, and no dead cells were observed. Figure 6 represents the image panel of the live–dead assay performed to evaluate the cell viability for all the hydrogel-treated plates. Similar findings have been reported in a previous study [38], indicating the effectiveness of plant bioactive-combined nanomaterials as a potential wound dressing material.

### 2.9. MIC Determination of the Hydrogels

Microbial contamination can lead to prolonged inflammation, increased attraction of pathogens that ultimately interrupt tissue regeneration, and increased collagen secretion, leading to the formation of various types of scars. Therefore, determining the antimicrobial effects of hydrogels against these microorganisms highlights the significant importance of wound care management. The antimicrobial effects of all the hydrogels were determined against *E. coli* and *S. aureus*.

**Figure 7:**
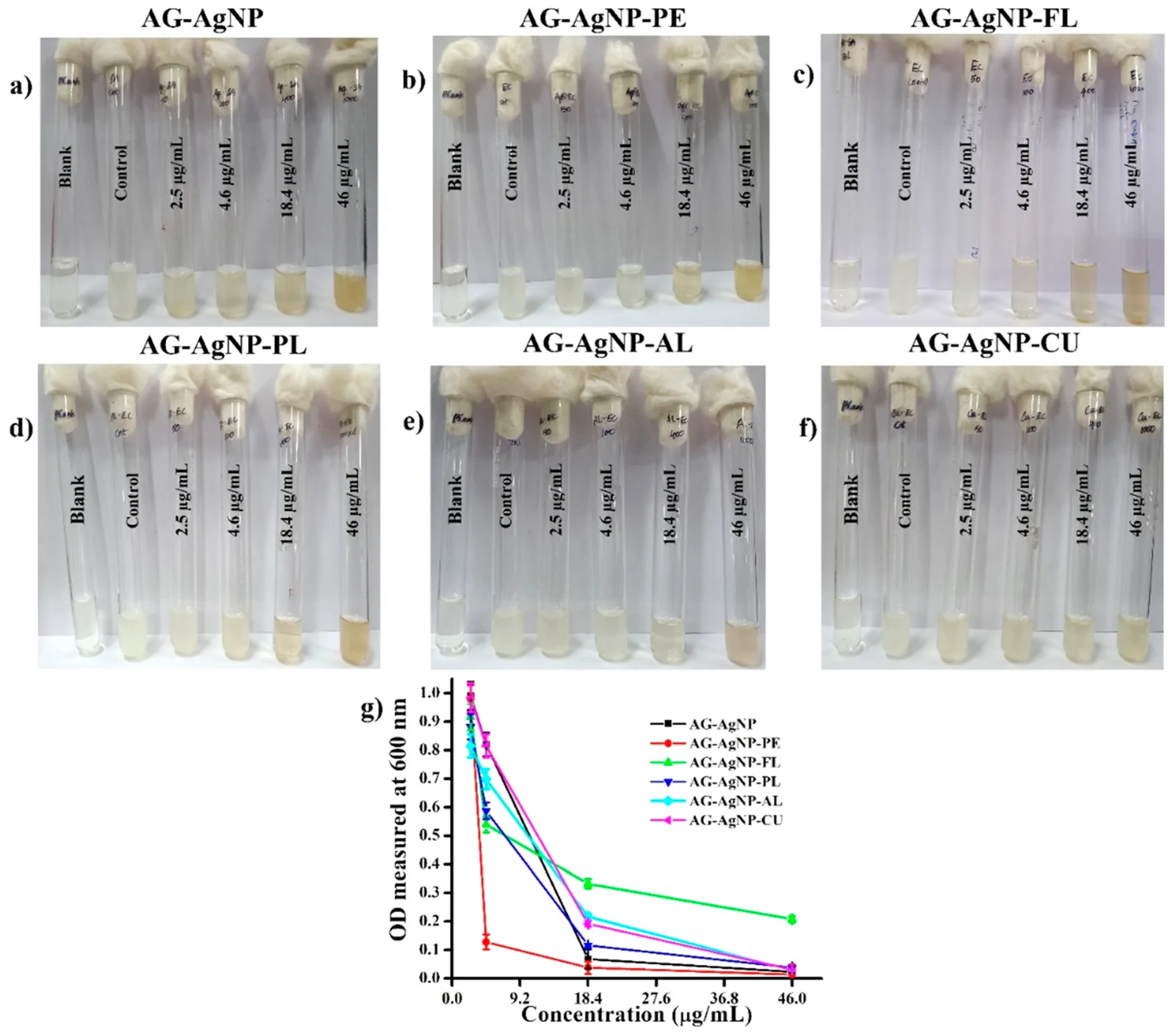
MICs determined for all the hydrogels treated with different concentrations against *E. coli*. (**a**) MIC tubes treated with AG-AgNP after 24 h, (**b**) MIC tubes treated with AG-AgNP-PE after 24 h, (**c**) MIC tubes treated with AG-AgNP-FL after 24 h, (**d**) MIC tubes treated with AG-AgNP-PL after 24 h, (**e**) MIC tubes treated with AG-AgNP-AL after 24 h, and (**f**) MIC tubes treated with AG-AgNP-CU after 24 h. (**g**) MIC data represented in a graphical form.

**Figure 8:**
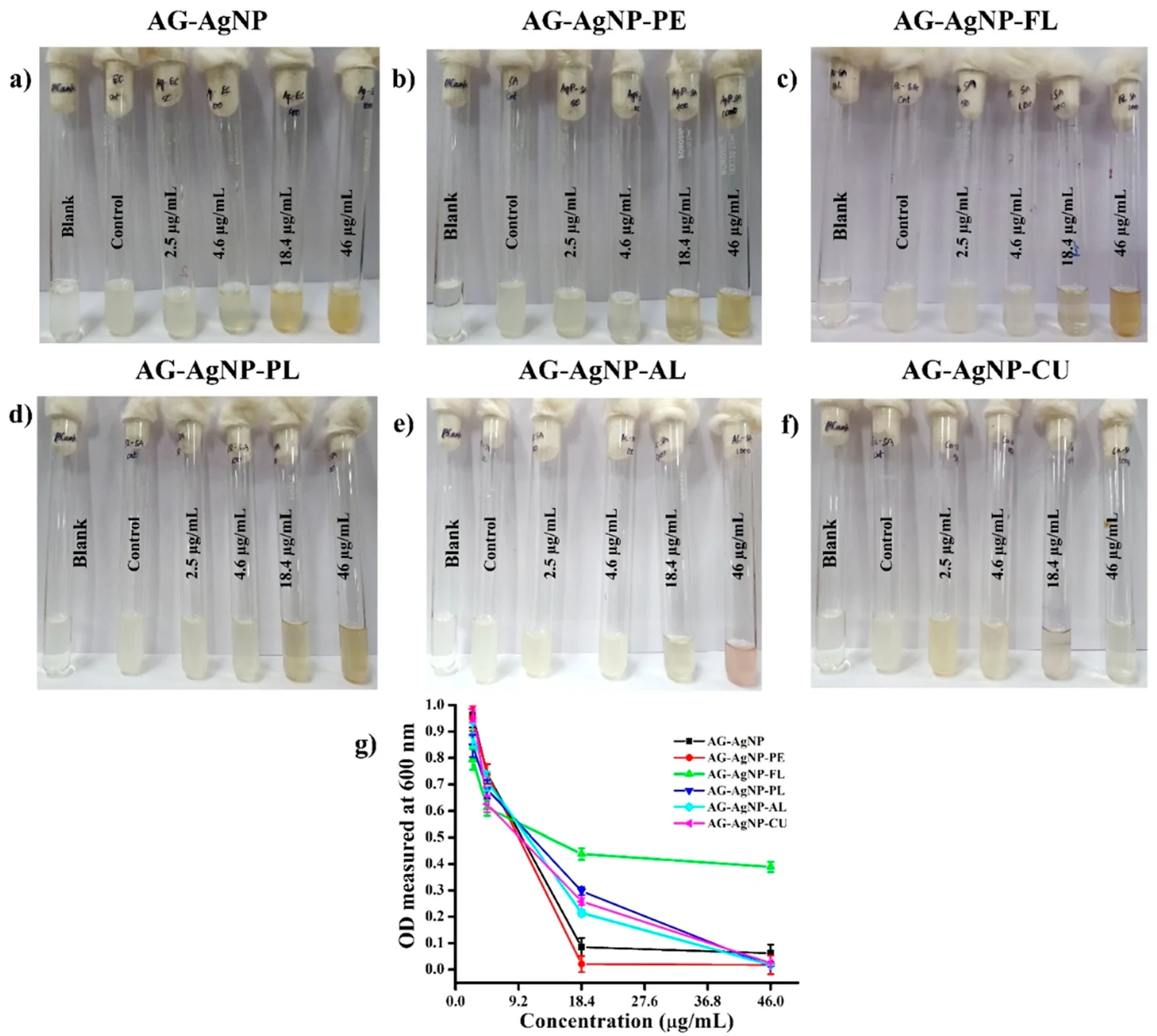
MICs determined for all the hydrogels treated with different concentrations against *S. aureus*. (**a**) MIC tubes treated with AG-AgNP after 24 h, (**b**) MIC tubes treated with AG-AgNP-PE after 24 h, (**c**) MIC tubes treated with AG-AgNP-FL after 24 h, (**d**) MIC tubes treated with AG-AgNP-PL after 24 h, (**e**) MIC tubes treated with AG-AgNP-AL after 24 h, and (**f**) MIC tubes treated with AG-AgNP-CU after 24 h. (**g**) MIC data represented in a graphical form.

Similarly, the MIC was determined for all the hydrogels, and it was observed that the hydrogels AG-AgNP and AG-AgNP-PE exhibited inhibition at a concentration of 18.4 µg/mL, whereas the other hydrogels, AG-AgNP-PL, AG-AgNP-AL, and AG-AgNP-CU, showed inhibition at a concentration of 46 µg/mL for both microorganisms. Figure 7 and Figure 8 show the MIC evaluated against the microorganisms *E. coli* and *S. aureus*.

These findings highlight that plant extracts possess superior antimicrobial effects, which, when combined, enhance complete pathogen inhibition activity. Remarkably, compared to the hydrogel AG-AgNP, the AG-AgNP-PE hydrogel demonstrated greater inhibition against both *E. coli* and *S. aureus*, reflecting its role in promoting wound healing by completely inhibiting the growth of the pathogens. As reported in the previous study, the plant extracts-infused hydrogel promotes enhanced antimicrobial activity by inhibiting the growth of pathogens, thus promoting rapid wound healing [42]. The developed hydrogel AG-AgNP-PE potentially inhibited the pathogens and promoted enhanced antimicrobial activity suitable for the promotion of a healing environment.

The above studies demonstrate that the developed AG-AgNP-PE possesses high antimicrobial, antioxidant, biocompatibility, and nontoxic properties, compared to other hydrogels. Hence, based on these promising results, further studies were performed to evaluate the potential of AG-AgNP-PE to promote scar-free wound healing in comparison with the AG-AgNP hydrogel.

### 2.10. Antibiofilm Activity

The biofilm assay was performed to investigate the capacity of the hydrogels to prevent bacterial biofilm formation. Both hydrogels exhibited superior antibiofilm activity by effectively inhibiting bacterial colonization. The hydrogel AG-AgNP-PE showed the highest biofilm inhibition percentages, 97% and 96%, against *E. coli* and *S. aureus* after 96 h, respectively. Meanwhile, the hydrogel AG-AgNP showed an inhibition percentage of 95% and 94% in *E. coli* and *S. aureus* at the same time point.

**Figure 9:**
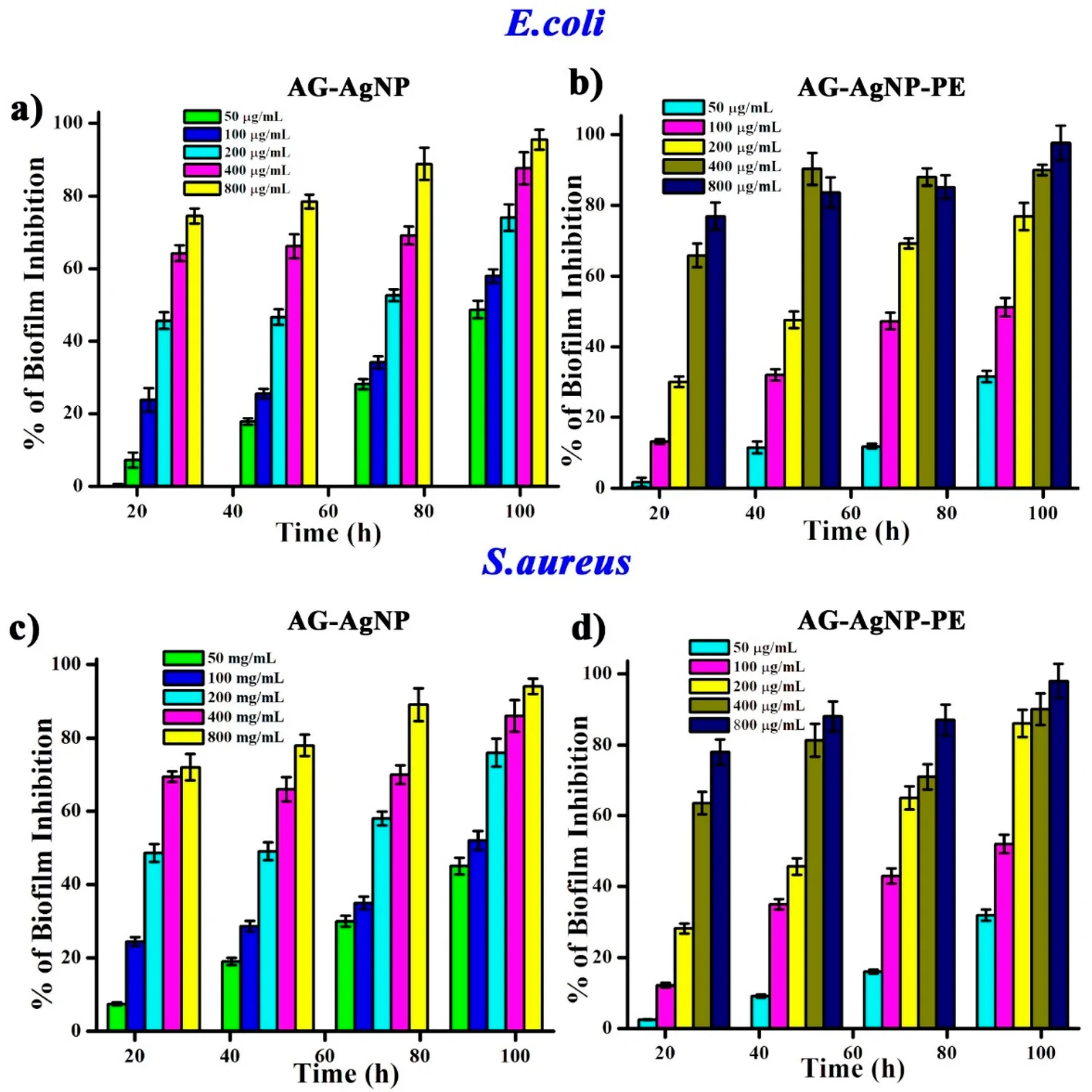
Graphical images of antibiofilm activity of the developed hydrogels against *E. coli* and *S. aureus*. (**a**,**b**) Percentage of biofilm inhibition possessed by the hydrogels AG-AgNP and AG-AgNP-PE against *E. coli*, respectively. (**c**,**d**) Percentage of biofilm inhibition by the hydrogels AG-AgNP and AG-AgNP-PE against *S. aureus*, respectively.

These findings indicate that both hydrogels possessed strong and superior antibacterial activity and antibiofilm properties that can effectively prevent bacterial colonization and reduce inflammation at the wound site. The enhanced performance of the hydrogel AG-AgNP-PE suggests that the synergistic effect of plant bioactives, along with silver nanoparticles, promotes the healing of wounds by creating a favorable environment for rapid and scar-free wound healing. Figure 9a,b illustrates the antibiofilm activity exhibited by the hydrogels AG-AgNP and AG-AgNP-PE against *E. coli*, whereas Figure 9c,d shows the antibiofilm activity against *S. aureus*. These findings were also supported by the previously reported study, where the silver nanoparticle combined with phytochemicals was capable of attaining the complete inhibition of bacteria [43].

### 2.11. Zebrafish Embryo Assay

Due to advantages such as rapid growth, size, transparency, and ease of monitoring, the zebrafish embryo is an excellent in vivo model for toxicity assessment. Although the assessments performed using the scratch assay and the antimicrobial assay suggest that it provides the essential wound healing properties, this model was further used for determining the toxicity effects of the developed hydrogels AG-AgNP and AG-AgNP-PE.

**Figure 10:**
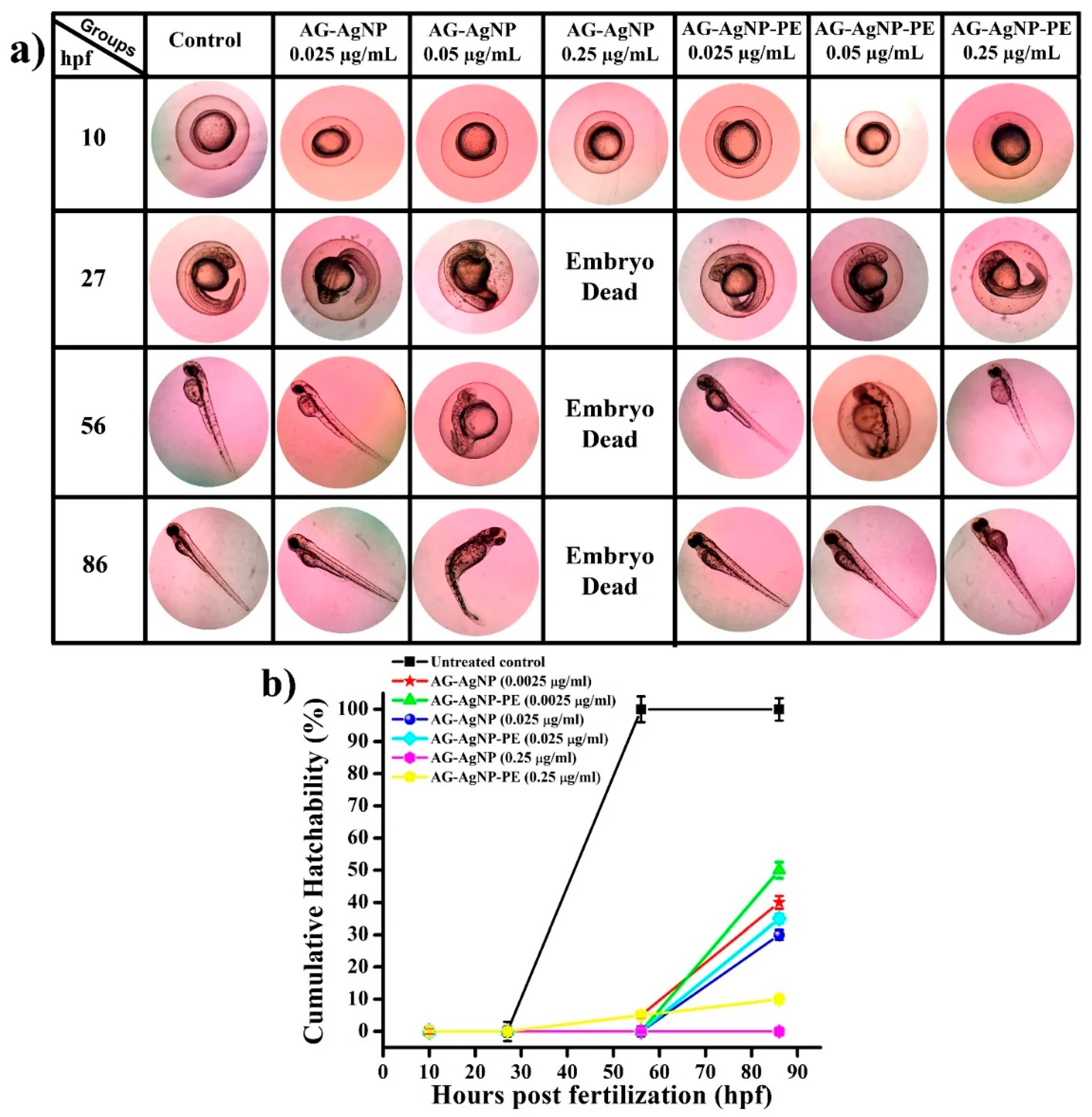
Toxicity assessment of the hydrogels on zebrafish embryos. (**a**) Image panel showing the morphological changes at 86 hpf and photos of embryos treated with hydrogels. (**b**) Cumulative hatchability percentages.

The toxicity assessment was performed, and the toxic effects of the hydrogel were observed. Changes in hatching, body movements, and appearance were noted and are shown in Figure 10a. The embryos were treated with various concentrations of the hydrogels AG-AgNP and AG-AgNP-PE. The embryos treated with 0.025, 0.05, and 0.25 µg/mL of AG-AgNP-PE showed usual growth parallel to untreated control embryos, while the embryos treated with AG-AgNP at the same concentrations exhibited delayed hatching at 56 hpf, with abnormalities in growth and morphological changes in 86 hpf, likely due to the toxic effects, as reflected by embryo death. Further, the cumulative hatchability percentage (%) was also determined for both hydrogels up to 86 hpf and plotted as a graph (Figure 10b).

The graph clearly shows that hatchability was higher in both concentrations of AG-AgNP-PE than AG-AgNP, even at 86 hpf. Delayed hatching and morphological changes were also observed for the AG-AgNP group at a medium concentration (0.05 μg/mL), whereas only delayed hatching was observed for AG-AgNP-PE at 56 hpf in 0.05 μg/mL; however, complete hatching in the same concentration was observed at 86 hpf, with no abnormalities observed in untreated embryos. This clearly indicates that the addition of plant extracts with AG-AgNPs decreased the toxicity and increased its biocompatibility, contributing to potential biomedical applications, as supported by the previously reported study [44,45].

### 2.12. In Ovo Model

**Figure 11:**
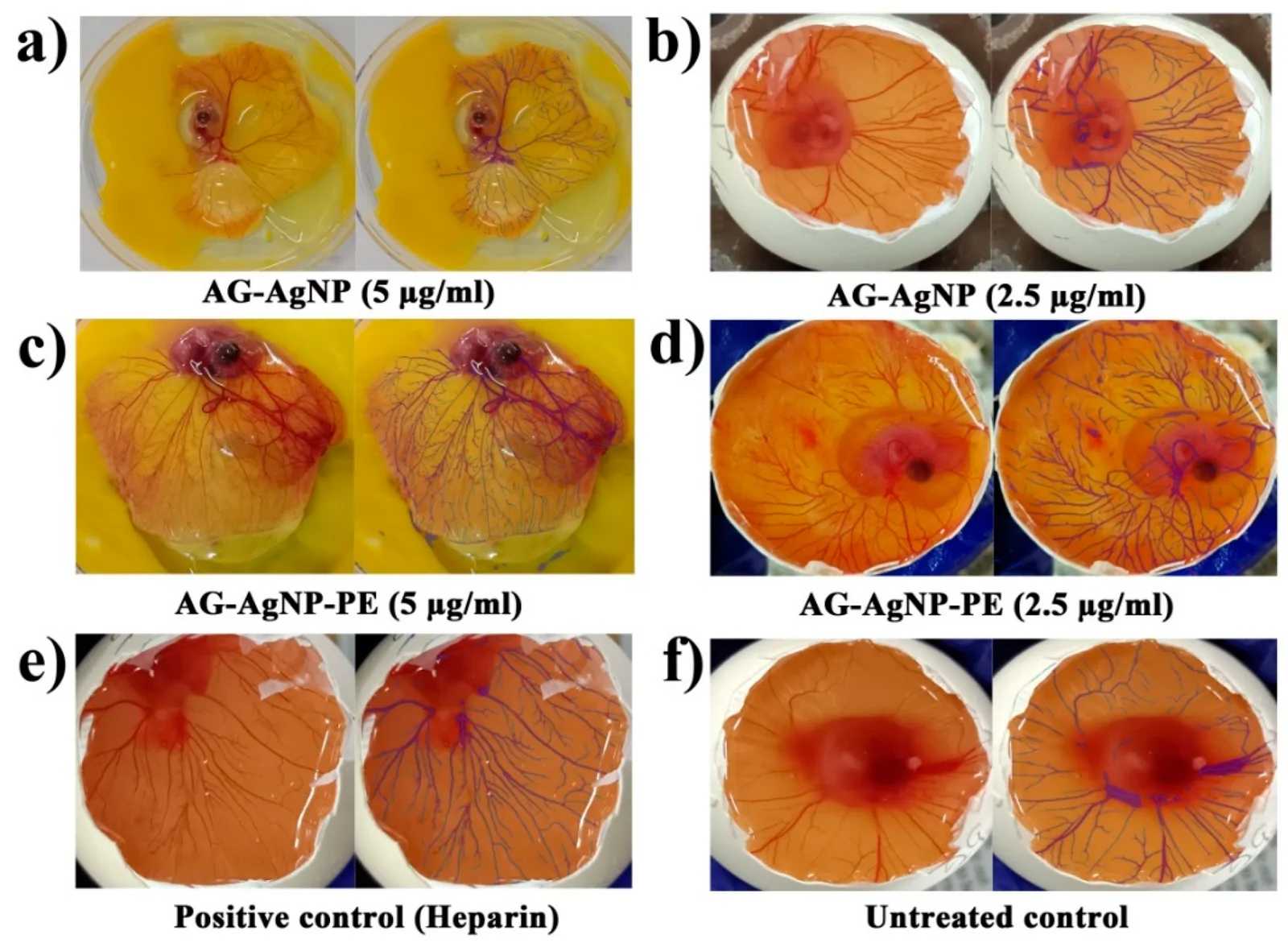
Angiogenic activity evaluated in the CAM membrane. (**a**,**b**) AG-AgNP-treated CAM membrane in both higher and lower doses, (**c**,**d**) AG-AgNP-PE-treated CAM membrane with both higher and lower doses, (**e**) positive control, and (**f**) untreated control.

The CAM assay is important as it replicates the process of vascular growth in wound healing, called angiogenesis. The CAM assay identifies and evaluates the angiogenic activity promoted by the prepared hydrogels. Angiogenesis occurs only when the tissue is packed with oxygen, nutrients, immune cells, and growth factors because the compounds promote the production of new blood vessels. Further, this promotes the synthesis of collagen, fibroblast cells, granulation tissue, and tissue regeneration, leading to complete wound closure. The hydrogel AG-AgNP-PE-treated eggs showed new small blood vessel formation, which was observed both in higher and lower concentrations (Figure 11c,d). However, less blood vessel formation was observed in both AG-AgNP-treated eggs (Figure 11a,b), indicating less efficient promotion of angiogenic activity, as slightly evidenced in the untreated control (Figure 11f). The pro-angiogenic activity observed in AG-AgNP-PE indicates the synergistic effects between the plant extracts and the other compounds present, as observed in heparin-treated eggs (Figure 11e).

**Figure 12:**
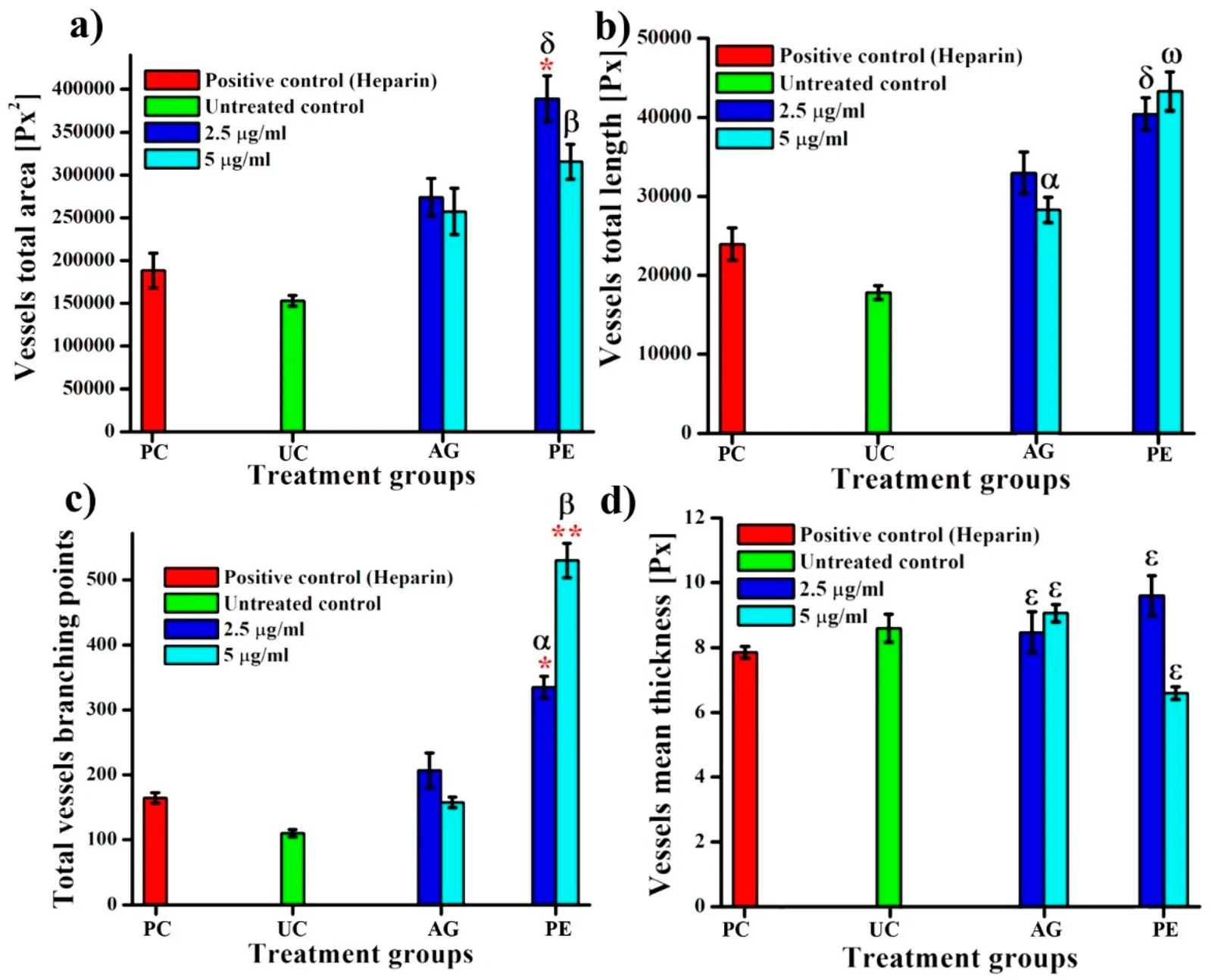
Illustrates the quantitative analysis of angiogenic activity, where (**a**,**b**) represents vessel total area and vessel total length, whereas (**c**,**d**) represents the vessel branching points and mean thickness. The results are expressed as mean ± SD, and statistical significance levels were calculated and compared with the control and between the groups. Comparisons with the untreated control are indicated as ε (*p* < 0.001), δ (*p* < 0.005), ω (*p* < 0.01), β (*p* < 0.05), and α (*p* < 0.1). Likewise, the comparisons with treatment groups are indicated as * *p* < 0.1 and ** *p* < 0.05, respectively.

Therefore, the findings validate that the hydrogel AG-AgNP-PE significantly promotes angiogenic activity in the in ovo model. Later, the other parameters like vessel area, vessel length, vessel branching point, and mean thickness were calculated and plotted in a graphical format to support the above evidence (Figure 12a–d).

The quantitative analysis showed higher vessel total area and mean thickness in both the low- and high-dose AG-AgNP-PE-treated groups compared to the positive control. Likewise, the vessel branching points and vessel length were observed to be superior in the high-dose AG-AgNP-PE-treated group compared to the lower-dose group, indicating the potential activity of angiogenic development. However, all parameters were noted to be less effective in the AG-AgNP-treated group compared to AG-AgNP-PE and the positive control groups. The high vascular production noted in AG-AgNP-PE-treated groups suggests its ability to promote blood vessel formation in biological systems, which is essential for enhanced tissue regeneration and repair. These findings were consistent with a previously reported study [46], which confirms that the developed hydrogel AG-AgNP-PE enhances angiogenic activity, leading to rapid healing and deposition of tissues.

### 2.13. Determination of Scar-Free Wound Healing in Wistar Rats

**Figure 13:**
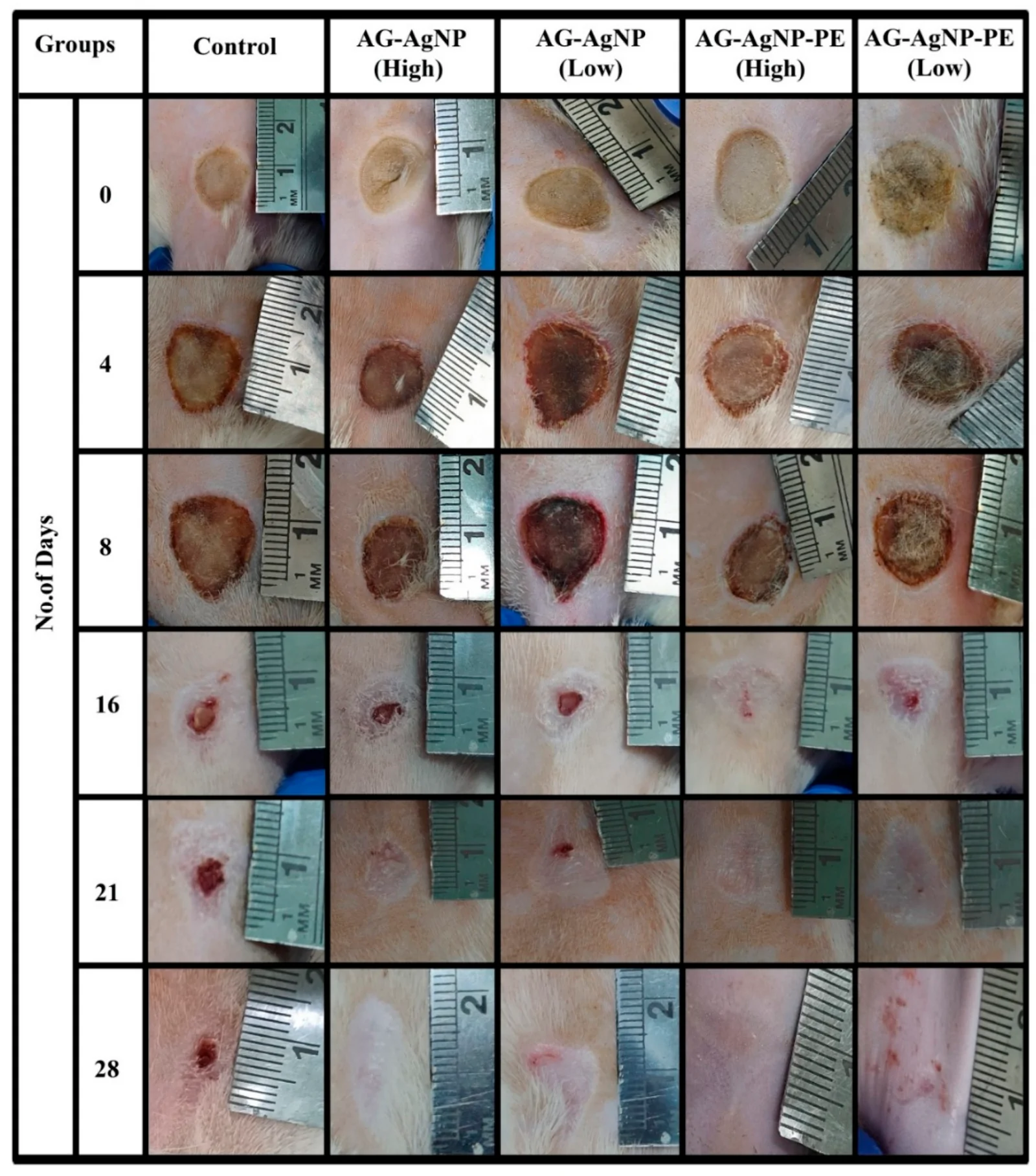
Twenty-eight-day scar-free wound healing study performed on an in vivo animal model.

The scar-free wound healing study was performed to observe the effect of the prepared hydrogels on Wistar albino rats, as it provides direct evidence of a wound healing material enhancing the healing mechanism in a living system that consists of various biological processes. The scar-free wound healing study was performed for 28 days on an animal model with different doses of the prepared hydrogels (30 mg/kg and 100 mg/kg). The negative control was treated with saline. The wound was created for all the animals on the same day, and treatment was provided regularly. Group 1 represents the animals treated with saline, group 2 was treated with AG-AgNP at a higher dose (100 mg/kg), whereas group 3 was treated with AG-AgNP at a lower dose (30 mg/kg). Similarly, groups 4 and 5 consisted of animals treated with AG-AgNP-PE at high (100 mg/kg) and low (30 mg/kg) doses, respectively. Figure 13 shows the gradual healing of wounds and scar-free healing in the animal model.

The animals treated with a higher dose of hydrogel AG-AgNP-PE showed superior healing by the 16th day compared to control-treated animals and AG-AgNP groups, indicating the post-inflammatory and early proliferation phases. On the 21st day, we clearly observed complete wound healing with mild scar formation in the AG-AgNP-PE-treated groups, indicating the early remodeling phase. Meanwhile, the wound was still observed in the control group as well as the high-dose AG-AgNP-treated group, whereas a mild wound was observed in the low-dose AG-AgNP-treated group. The hydrogel AG-AgNP-PE exhibited scar-free wound healing at both the higher and lower doses on the 28th day, which indicates complete healing and normal tissue regeneration. These findings suggest no excess collagen production or fibroblast secretion. However, the groups treated with a high dose of AG-AgNP showed complete healing with a scar, whereas mild wounds and scars were observed in the low-dose group. In contrast, the unhealed wound was still observed in the control-treated group, indicating a delayed healing process. Therefore, the image panel reflects the capacity of the prepared hydrogels to promote scar-free wound healing. These findings demonstrate that AG-AgNP-PE has a higher ability to promote scar-free and rapid wound healing compared to AG-AgNP. This proves that the synergistic effects of plant extracts are most important in promoting natural, faster, and scar-free healing [47].

### 2.14. Histopathological Analysis

**Figure 14:**
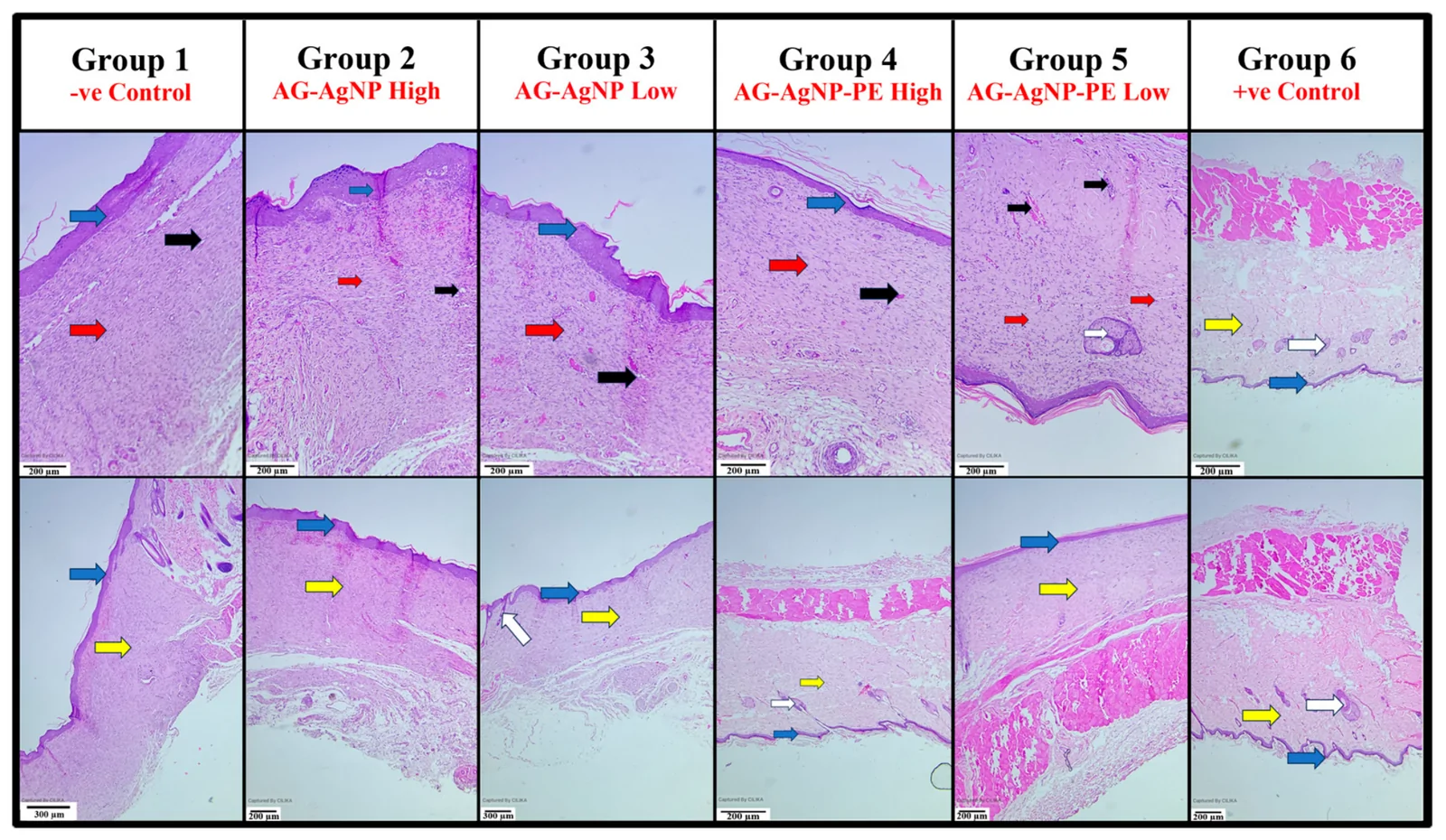
Representative image panel of the H&E-stained histological sections of wound tissues excised after treatment with saline, no saline, and hydrogels.

Scar-free wound healing can be claimed if the histopathological analysis of normal skin (taken from another area where there is no scar created or healed) and the healed skin are similar. This will justify that the traits of healed skin using AG-AgNP-PE are similar to normal skin, where no wound was created. To perform this analysis, we have performed histopathological analysis of the healed skin mediated by normal saline (G1), AG-AgNP at a high dose (100 mg/kg) (G2), AG-AgNP at a low dose (30 mg/kg) (G3), AG-AgNP-PE at a high dose (100 mg/kg) (G4), AG-AgNP-PE at a low dose (30 mg/kg) (G5), and normal skin excised from any other part of the body (G6), named as the positive control (+ve control). The histopathological analysis was done to further confirm the above results. The healed skin area and normal skin were excised and stained using H&E staining. The stained sections were examined and photographed at 40× magnification. Table 3 indicates the histological changes observed in the regenerated tissues. Figure 14 illustrates the histopathological images of the stained tissues treated with both hydrogels and the control. The histopathological observation indicates that the hydrogel AG-AgNP and AG-AgNP-PE were nontoxic and did not induce any systemic damage. Furthermore, the AG-AgNP-PE hydrogel exhibited enhanced and superior healing, as evidenced by an increase in cellular organization and ECM matrix, denoting healthy tissue deposition as reported in a previous study [48]. The histological parameters of the healed skin post-treatment with AG-AgNP-PE at high and low doses were similar to those of normal skin, indicating that tissue regeneration was normalized, similar to that of normal skin. The metabolic cues in the tissue microenvironment provided by the AG-AgNP-PE hydrogel encouraged the local stem cells to regenerate normal skin layers during healing. Further confirmation of scar-free wound healing was obtained by estimating the growth factors, such as VEGF and PDGF, which are explained in the following sections.

### 2.15. Organ and Body Weight

Following the histological evaluation, the biocompatibility and potential toxicity of the hydrogels were studied by monitoring the organ weight and body weight of the treated animals. Though the treatment was provided on the dorsal wound area, the parameters were analyzed to identify and evaluate any possible systemic changes that occurred. The parameters were noted at the same time intervals as the wound healing study, where we observed no significant changes between the groups and the control. Table S1 shows the average relative body weight of the hydrogel- and saline-treated groups, whereas Table S2 shows the relative organ weight of the same groups as above. The absence of any significant difference indicates that the developed hydrogels did not induce any systemic toxicity or abnormal effects. The normal physiological functions of the hydrogels indicate that the developed hydrogels act as a potential biosafety wound dressing model that possesses robust wound healing properties. Similar findings were reported in previous studies, indicating that the biopolymer-based wound dressings with steady body and organ weight confirm the in vivo biocompatibility [49].

### 2.16. Assessment of Hematological Parameters

The hematological parameters were analyzed to assess the systemic toxicity and biocompatibility of the prepared hydrogels. The parameters were studied using the blood of the animals treated with the hydrogels. The blood was withdrawn on the 7th and 28th days. The parameters tested are listed and summarized in Table 4 and Table 5, respectively. There was no significant difference observed between the hydrogels and the control group on the 7th and 28th days. These findings showed no changes in parameters and fell within physiological limits, indicating that the treatment with prepared hydrogels did not provide any toxicity or changes in systemic responses, concluding the safety and biocompatibility of the hydrogels [49].

### 2.17. Growth Factor Analysis—VEGF and PDGF

**Figure 15:**
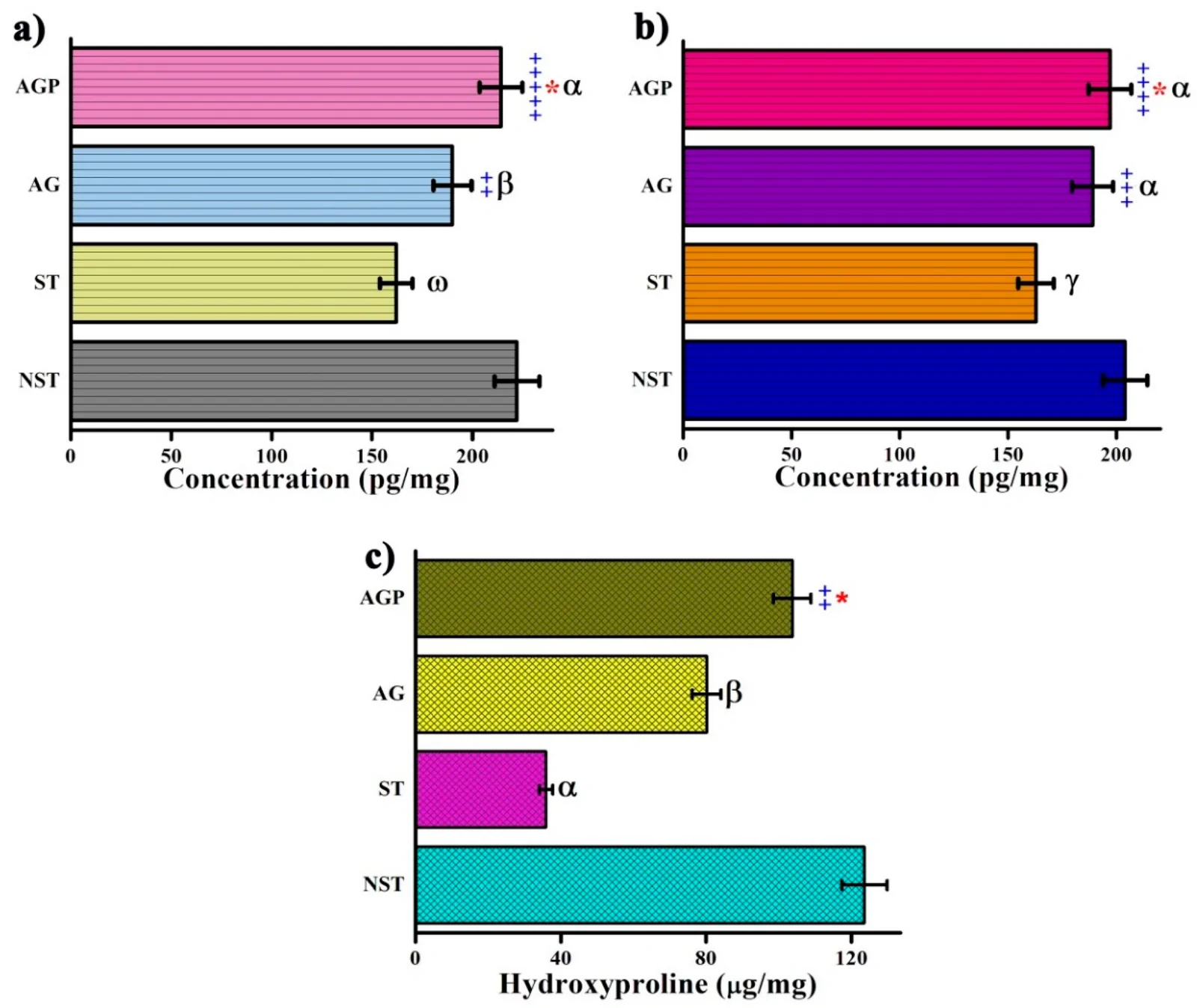
Levels of growth factors and collagen measured in the excised wound tissues. (**a**,**b**) Levels of VEGF and PDGF growth factors exhibited in different groups. Statistical significance for growth factor analysis compared with the positive control is indicated as ω (*p* < 0.005), β (*p* < 0.025), α (*p* < 0.05), and γ (*p* < 0.01). Comparisons with the negative control are indicated as ++ (*p* < 0.05), +++ (*p* < 0.025), ++++ (*p* < 0.01), +++++ (*p* < 0.005), while comparisons between treatment groups are indicated as * *p* < 0.1, respectively. Similarly, (**c**) shows the collagen level deposited in the excised tissues of the respective groups. The level of statistical significance of collagen compared with the positive control is indicated as β (*p* < 0.05) and α (*p* < 0.01). Comparisons with the negative control and treatment groups are indicated as ++ (*p* < 0.025) and * *p* < 0.10, respectively.

Following the in vivo studies, the growth factor analysis was conducted to provide molecular evidence that the treatment promotes new tissue formation and that the healing is scar-free. The growth factors promote healing by increasing oxygen supply, essential cell migration and proliferation, new blood vessel formation, and high vascular tissue formation, leading to superior angiogenesis and enhanced healing. If these growth factors are expressed at the same level in AG-AgNP-PE-treated skin and normal skin (without any wound or scar), we can speculate that the hydrogel has induced scar-free wound healing. Growth factor analysis, such as VEGF and PDGF, was performed for both prepared hydrogels (high dose) and controls (saline-treated healed skin and normal skin without any scar or healing). Saline-treated healed skin is named the control group (ST), and the skin excised from a healthy part without any scar or healing is named the positive control (NST). The analysis demonstrated that VEGF and PDGF were superiorly expressed in the AG-AgNP-PE-treated group, similar to the positive control group (NST). However, both growth factors were weakly expressed in the control group (ST) compared to AG-AgNP. Therefore, the findings indicate that the superior expression of the growth factors VEGF and PDGF was promoted by the incorporation of the plant extracts, which stimulate the process of angiogenesis by increasing the proliferation and migration of endothelial cells and fibroblast cells. This ultimately promotes the growth of new blood vessels, which increases the oxygen and nutrient supply to the regenerating tissues and increases the deposition of new ECM matrix, collagen production, and tissue remodeling. The synergistic effect of AG-AgNP-PE likely modifies the wound microenvironment by minimizing inflammation and oxidative stress, which activates the growth factor-mediated signaling pathways. Hence, the upregulation of both growth factors helped in rapid tissue regeneration, increased vascular growth, and an organized ECM matrix, leading to scar-free healing in AG-AgNP-PE treated wounds. The results are expressed as mean ± SD, where the statistical significance level was calculated and compared with NST and ST, and between treatment groups, as represented in Figure 15a,b. Similar findings were reported, in which a plant bioactive-based hydrogel loaded with polymeric nanoparticles enhanced rapid healing by promoting essential growth factors involved in wound healing [50].

### 2.18. Hydroxyproline Analysis

The hydroxyproline assay measures the levels stimulated by the biosynthesis of collagen, which promotes wound repair by enhancing quality, supporting well-organized ECM deposition rather than rapid wound closure. The collagen level deposited in the tissue of NST was higher than in the ST and AG-AgNP-treated groups. The AG-AgNP-PE group showed a similar collagen level to that observed in the NST group. The collagen levels of each group are represented in Figure 15c. The collagen level deposited in the AG-AgNP-PE group was higher compared to that in the AG-AgNP and saline-treated groups. The overall data obtained from wound healing, histopathology, growth factor analysis, and hydroxyproline analysis prove that the AG-AgNP-PE hydrogel not only promotes wound healing but also promotes high collagen formation, resulting in scar-free wound healing. The expressed levels were represented as mean ± SD. The statistical level of significance was obtained by comparison with positive and negative controls and with treatment groups. Studies have reported that phyto-infused hydrogels or nanomaterials demonstrate excellent biocompatibility, controlled release, and sustained antimicrobial and antioxidant activities. They also suggested that the developed nanomaterial promotes collagen deposition by stimulating vascular responses and regulating macrophage production [51].

## 3. Conclusions

In the present study, a series of alginate, gelatin-based hydrogels incorporated with silver nanoparticles and bioactive plant extracts was developed successfully, and scar-free wound healing was evaluated in an animal model. Among the six developed hydrogels, the hydrogel AG-AgNP-PE, i.e., the hydrogel combined with all the plant extracts, demonstrated the greatest overall performance. The incorporation of numerous phytochemicals significantly enhanced the biological activity of the hydrogel by providing increased antimicrobial, antioxidant, anti-inflammatory, and pro-angiogenic activity that stimulated the controlled release of silver nanoparticles. The obtained synergistic effects of the plant extracts combined hydrogel rapidly accelerated scar-free wound healing by promoting superior wound closure, enhanced biocompatibility, improved angiogenesis, increased deposition of collagen and ECM, and superior tissue remodeling compared with the nanomaterial-based hydrogel alone. These findings point out that integrating various plant bioactives into a silver nanoparticle-loaded alginate/gelatin hydrogel can effectively overcome the limitations associated with the nanomaterial alone by controlling oxidative stress, minimizing inflammation, improving biocompatibility, and promoting balanced tissue formation. Instead of depending only on antimicrobial and antioxidant properties, the developed hydrogel AG-AgNP-PE actively modulates the various phases of wound healing, ultimately leading to scar-free healing. Overall, the present study provides a simple, highly biocompatible, and multifunctional hydrogel platform with major prospects for advanced wound care management. The aesthetic value of burns is enhanced due to the scar-free healing effect. The developed formulation represents a promising strategy for the treatment of burn wounds, and further studies involving molecular investigations and clinical studies exhibit its translational efficacy into clinical applications.

## 4. Materials and Methods

### 4.1. Materials

The chemicals such as Luria broth, Dulbecco’s modified Eagle medium solution, Antibiotic & antimycotic solution, Sodium alginate, and MMT (3-4,5-Dimethylthiazol-2-yl)-2,5-Diphenyltetrazolium Bromide were purchased from HiMedia, India. The Fetal Bovine Serum and Gelatin Bloom 175 were purchased from SIGMA and Gibco, USA. Glutaraldehyde, acetone, curcumin, silver nitrate, dimethyl sulphoxide, and calcium chloride were procured from SRL and RANKEM, India. Similarly, the *Calendula* flower powder was purchased online, aloe vera was purchased from the market, and plantain was procured from the Chettinad Organic Garden, Chennai, India. The Chinese hamster fibroblast cell line (V79) was purchased from NCSS, Pune, India.

### 4.2. Preparation of the Natural Extracts

The locally obtained natural materials, aloe vera and plantain peel, were readily washed and peeled. The plantain peel was shade-dried for a week and powdered. Aloe vera pulp was washed and stirred to obtain the natural extract, whereas the *Calendula officinalis* flower and curcumin extract were prepared following the usual procedure. The detailed preparation was described in the following literature [52].

### 4.3. Preparation of Hydrogels

The alginate and gelatin solutions were prepared individually by dissolving 1 g and 1.53 g in water at 50 °C with stirring. Once fully dissolved, acetone was rapidly introduced to the gelatin solution and mixed for 10 min to allow the particles to settle. The liquid layer was decanted, and the settled layer was suspended in distilled water and agitated at 50 °C for 10 min. The alginate composition was formed by mixing 2 mL of the solution into the gelatin suspension and maintaining it for the same period. Further, 1% silver nitrate was added, mixed, and exposed to ultraviolet light for 1 h under continuous stirring. In order to crosslink and form gelation, 1% glutaraldehyde was added and stirred, and then 3.1 mL of distilled water or individual extracts was added. Later, 290 µL of calcium chloride was added and mixed for 30 min. The hydrogel AG-AgNP was prepared and refrigerated at 4 °C until required [52].

The same protocol was followed with minor modifications for the preparation of the remaining hydrogels. For the preparation of AG-AgNP-PE, 3.1 mL of distilled water was replaced by 1000 µL of aloe vera, plantain peel, and *calendula* flower extract, along with 100 µL of curcumin solution. Likewise, for the preparation of AG-AgNP-PL, AG-AgNP-FL, AG-AgNP-AL, and AG-AgNP-CU, 3.1 mL of each extract was added individually, replacing the distilled water.

### 4.4. Characterization of the Hydrogels

UV-visible spectroscopy, dynamic light scattering (DLS), zeta potential analysis, and Fourier-transform infrared spectroscopy were all used to characterize the hydrogels. The hydrogel samples were prepared and properly diluted with distilled water before measurement. The UV-visible absorption spectra were measured with a Shimadzu (Kyoto, Japan) UV-1900 spectrophotometer, and a Malvern NanoZS90 (Malvern, UK) particle size analyzer was used to determine the hydrodynamic diameter and zeta potential of the particles to assess the colloidal properties. FTIR spectra were collected using a Bruker Alpha FTIR spectrophotometer ranging from 4000 to 400 cm^−1^ to identify the functional groups and chemical interactions involved in hydrogels. Surface morphology was measured using a scanning electron microscope (JSM-IT800, JEOL Ltd., Tokyo, Japan).

### 4.5. Release Kinetics

The release kinetics provide crucial information about the hydrogel’s properties, such as its ability to achieve prolonged and controlled release, specifically in the wound environment, thereby enhancing the healing capacity of the wound. A hydrogel (2 mL) was added to a dialysis bag and immersed in 30 mL PBS (pH = 7.4) in a glass beaker under stirring conditions. The stirring was conducted for 48 h, with sampling performed at every 30 min interval up to 8 h, then 24 h, and subsequently every 4 h until 36 h and 48 h. At each time point, 2 mL of released medium was withdrawn and replaced with fresh PBS in the glass beaker. Finally, the optical density was measured at 416 nm, and the data were represented as a graphical representation. The cumulative release percentage was calculated following the formula given below [53]

$$Cumulative\;release\;\left(\%\right)=\frac{OD\;measured\;at\;time\;}{Total\;Drug\;OD}\times 100\;$$

### 4.6. Swelling Assay

The swelling assay was performed to evaluate the fluid uptake capacity of the hydrogels by following the procedure described with minor modifications. Phosphate-buffered saline (PBS) was used as the swelling medium, and 100 mg hydrogels were weighed and designated *W*_1_. Later, 2 mL of PBS (pH = 7.4) was added to a 35 mm Petri dish and incubated at room temperature; at intervals of 60 min, the medium was removed, and the plate was dried for some time. Later, the dried plate was weighed to measure the swollen gel, which was designated *W*_2_. Likewise, the weight was measured at every 60 min interval for 9 h and then measured at 24 h. Finally, the percentage of swelling capacity of the hydrogels AG-AgNP and AG-AgNP-PE was represented in a graphical format. The swelling percentage calculation was performed for both the hydrogels using the following formula [54].

$$Swelling\;\%=\frac{W_{2}-W_{1}}{W_{1}}\times 100$$

where *W*_2_—weight of swollen hydrogel; and *W*_1_—weight of dried hydrogel.

### 4.7. DPPH Assay

The DPPH assay was performed to evaluate the antioxidant property of the extracts and hydrogels, following slight modifications [55]. The extracts, including plantain, *Calendula* flower, aloe vera, and curcumin, and hydrogels, like AG-AgNP and AG-AgNP-PE, were utilized. The assay was carried out using two different doses: a high and a low dose. A 0.1 mM 2,2-diphenyl-1-picrylhydrazyl (DPPH) solution was prepared by dissolving it in methanol, and the solution was covered with aluminum foil. Treatment samples like aloe vera extract, calendula extract, plantain peel extract, curcumin extract, AG-AgNP, and AG-AgNP-PE were utilized at predetermined concentrations. Ascorbic acid was utilized as a positive control. The high dose consists of 500 µL of the extract mixed with 500 µL of methanol, whereas the low dose consists of 250 µL of the extract mixed with 750 µL of methanol. To all the test tubes covered with foil, 2 mL of the prepared DPPH solution was added one by one, and the initial OD (A_0_) was measured at 517 nm. Then the measured tube was incubated in the dark for about 30 min. Post-incubation, the final OD (*A*_30_) was measured at the same wavelength, 517 nm. Finally, the percentage of ROS scavenging activity was determined by following the formula given below,

$$ROS\;\%=\frac{A_{0}-A_{30}}{A_{0}}\times 100$$

where A_0_—initial OD measured at 517 nm; A_30_—final OD measured at 517 nm.

### 4.8. Determination of Cytotoxic Effect by MTT Assay

The cell viability assay was performed in the V79 cell line, where 10^4^ cells/well were seeded in a 48-well microplate containing complete medium. The study was carried out in triplicate, and the cells were incubated at 37 °C with 5% CO_2_ for 24 h. Later, all hydrogels were treated with different concentrations (0.5, 2.5, and 5 µg/mL) after filtering the hydrogels and diluting them with complete medium. The untreated wells were considered the control, and the plate was again incubated under the same conditions mentioned above. Post-treatment, MTT was added and incubated for 4 h, and later DMSO was added to dissolve the formazan crystals formed. Optical density was measured at 570 nm, and the % of cell viability was calculated using the formula below and represented as a figure [54].

$$Cell\;viability\;\left(\%\right)=\frac{OD\;of\;Treated\;cells}{OD\;of\;Untreated\;cells}\times 100$$

### 4.9. Determination of Cytotoxicity by Alamar Blue Assay

The Alamar Blue assay was performed similarly to the MTT assay with slight modifications. The assay was performed in the V79 cell line, where 10^4^ cells/well were seeded in a 48-well microplate containing complete medium. The study was carried out in triplicate, and the wells were incubated at 37 °C with 5% CO_2_ for 24 h. Later, all hydrogels were treated at different concentrations (0.5, 2.5, and 5 µg/mL) after filtering the hydrogels and diluting them with complete medium. The untreated wells were considered the control, and the plate was again incubated under the same conditions mentioned above. Post-treatment, the previous medium was replaced with 0.15 mg/mL of 10 vol.% Alamar Blue reagent and complete medium and incubated for 4 h; later, the color change was observed. Fluorescence intensity was measured using a Jasco FP-3800 plate reader by setting the excitation at 530 nm and emission at 584 nm. Hence, the % of cell viability was calculated using the given formula below, and the graphical representation was shown as a figure [9].

$$Cell\;viability\;\left(\%\right)=\frac{\;Fluorescence\;intensity\;of\;Treated\;cells\;at\;584\;\mathrm{nm}}{Fluorescence\;intensity\;of\;Untreated\;cells\;at\;584\;\mathrm{nm}}\times 100$$

### 4.10. Live–Dead Assay

The assay was carried out on the same cell line as before, with slight modifications [56]. The cells were seeded onto the sterile coverslip placed in a 35 mm Petri dish, comprising 2mL complete medium. The plates were incubated in a humidified 5% CO_2_ incubator for 24 h and treated with the different concentrations of all the hydrogels (0.5 and 5 µg/mL). After treatment, the medium was discarded, and the plates were incubated at 37 °C for 3 min with fresh staining solution containing 2 µL of Ethidium Bromide (1.9 mM stock), 2 mL PBS, and 5 µL of Acridine Orange (1.2 mM stock). Later, the coverslip was washed with PBS and placed onto the fluorescence microscope (Olympus BX51) stage to visualize the live and dead cells represented by green and red fluorescence, respectively. The dead cell measurement was done following the formula [52].

$$No.\;of\;Dead\;cells\;(\%)=\frac{Total\;no\;of\;Dead\;cells}{Total\;no\;\;of\;(Live+dead)\;cells}\times 100$$

### 4.11. Scratch Assay

The wound healing (scratch) assay was performed on the same cell line using 35 mm Petri dishes. Cells were seeded and incubated for 24 h, after which a scratch was made, and the cells were treated with different concentrations (0.5 and 5 µg/mL) of all the hydrogels. The treatment was continued for 72 h, with images captured at 40× magnification at regular intervals. Later, the wound closure area was matched, quantified, and represented as a graph [52].

### 4.12. Determination of Minimum Inhibitory Concentration

Inhibiting bacterial growth is one of the crucial properties in wound healing. Antimicrobial activity improves the rapid release of growth factors that promote the proliferation of essential cells, initiating complete wound healing. Hence, the antimicrobial effect was studied against the microorganisms *Escherichia coli* (*E. coli*) and *Staphylococcus aureus* (*S. aureus*) for all the hydrogels. The microorganisms were freshly sub-cultured and used for the study. The cultures were divided into 15 test tubes labeled with the corresponding hydrogels, positive control, or negative control. Twelve test tubes were marked as treatment groups, where 100 µL of both the cultures + 3900 µL of Luria broth + 1000 µL of individual hydrogels was added and incubated. Similarly, to 2 test tubes named as the negative control, 4900 µL of Luria broth + 100 µL of both the cultures were added individually and incubated for 24 h. The positive control tube contained 5000 µL of Luria broth. Later, all the test tubes were incubated in a shaking incubator at 37 °C for 24 h, and the photographs and the optical density were measured to determine the antimicrobial activity of the hydrogels. After the antimicrobial study, the MICs of all the hydrogels were determined following the method indicated below. A dilution series of hydrogels was carried out at different concentrations, such as 2.5, 4.5, 18.4, and 46 µg/mL, using 100 µL of culture. Later, the tubes were incubated for 24 h at 37 °C, and the optical density was measured at 600 nm after taking the photos. After that, the MIC of the hydrogels was determined and discussed [52].

### 4.13. Biofilm Assay

Further, the biofilm assay was performed to study the biofilm inhibition promoted by the hydrogels AG-AgNP and AG-AgNP-PE. The fresh microorganisms *E. coli* and *S. aureus* were sub-cultured in Luria broth and incubated for 24 h at 37 °C. The overnight-grown culture was diluted 1:100 into fresh broth (9 mL broth; 1 mL culture). The culture was distributed in 48-well plates and incubated for 6 h. The treatment was provided with different concentrations (2.5, 4.5, 9.2, 18.4, and 46 µg/mL) and incubated for 24, 48, 72, and 96 h, respectively. Post-treatment, the plates were washed thrice with distilled water by submerging to detach the dead bacteria. Later, 1% crystal violet solution (250 µL) was added, the plates were incubated for 15 min, and the solution was discarded by washing twice. A 30% Acetic acid solution (250 µL) was added to the plates and kept at room temperature for 15 min to solubilize the crystal violet. Finally, the optical density was measured at 580 nm with acetic acid as a blank. The percentage of biofilm inhibition was calculated by the formula below and represented as a graph [57].

$$Biofilm\;inbition\;\left(\%\right)=\frac{OD\;of\;Control-OD\;of\;Treated}{OD\;of\;Control}\times 100$$

### 4.14. Zebrafish Embryos Assay

The zebrafish embryo model was utilized to determine the cytotoxicity of the hydrogel due to its structure, growth period, and minimal observation. The cytotoxic effect of the hydrogels was determined by abnormalities and morphological changes witnessed in the embryos. The cytotoxic study was performed after IAEC ethical approval (IAEC 2/Proposal: 161/A.Lr: 123/Dt: 02.07.2024) was obtained from the Chettinad Academy of Research and Education. The study included about 80 embryos, of which 10 were divided into four groups of three different concentrations (0.5, 2.5, and 5 µg/mL) and a control group. The study was conducted in strict accordance with ARRIVE guidelines. Any abnormal morphological changes were observed before experimentation, and the embryos were seeded in 6-well plates, after which 10 h post-fertilization (hpf) photographs were taken. Following that, both hydrogels were treated and kept in the dark. Later, the morphological changes were observed and photographed at 28, 52, and 76 hpf using an optical microscope (Olympus CH20i). After observing the presence of any morphological changes, the cumulative hatchability percentage was calculated and plotted as a graph [58].

### 4.15. Evaluating the Angiogenesis Activity of the Hydrogels

Angiogenesis is one of the crucial properties in promoting wound healing. The chorioallantoic membrane assay (CAM) was performed to evaluate the biocompatibility of the hydrogels and their capacity to promote vascular growth, ultimately indicating the angiogenic activity that enhances wound healing [58]. Fertilized eggs were purchased and incubated before the experiment at 37 °C with 60% humidified conditions. Using 70% ethanol, the eggshells were wiped thoroughly; then, the CAM membranes were marked and the shell was cracked open with a vent of 1 cm^2^, carefully closed with gauze tape and incubated at 37 °C for 24 h. Meanwhile, the hydrogels were filtered by diluting them with PBS at concentrations of 2.5 µg/mL and 1.25 µg/mL in a sterile environment. The next day, the vent was reopened, a sterile filter disk was carefully dropped on the CAM membrane, and a treatment of 10 µL of AG-AgNP and AG-AgNP-PE was provided onto the disk. Then the vent was sterilized, resealed, and incubated. About 30 fertilized eggs were utilized for the study, with 5 eggs assigned to each group. The groups were classified as high and low concentrations, as mentioned in the following Table 6.

After the treatment, the eggs were again incubated for 5 days under the same conditions. Later, on the 5th day, all the eggs were opened, morphological observations were performed, and the vessel growth was photographed using an OPPO F26 mobile camera. Then the essential parameters were analyzed using IKOSA software (Version 5.3.0), and statistical analysis was performed using Student’s *t*-test, with the results presented in graphical form [58].

### 4.16. In Vivo Study Using Wistar Rat

#### 4.16.1. Wound Creation

The in vivo animal study was performed after IAEC clearance (IAEC3/Proposal: 172/A.Lr: 134/Dt: 13 December 2024) was received from the Chettinad Academy of Research and Education. The study was classified into 5 groups (n = 6), where

Group 1 was assigned as saline-treated (100 μL/kg);

Group 2 as AG-AgNP higher dose (100 mg/kg);

Group 3 as AG-AgNP lower dose (30 mg/kg);

Group 4 as AG-AgNP-PE higher dose (100 mg/kg);

Group 5 as AG-AgNP-PE lower dose (30 mg/kg).

Wistar Albino rats with a weight of 150–250 g were obtained from TANUVAS, Chennai. The ARRIVE guidelines were strictly followed for the study. Post-quarantine, the animals were segregated into their respective groups, and hair was removed to create a 10 mm wound on the dorsal neck region using a sterile, hot T-shaped steel rod. The wound was created by partially anesthetizing the animals using Isoflurane and was observed during the recovery period; the wound site was photographed and indicated as the 0th day. Later, the animals were treated with low and high doses of hydrogels and with saline as a negative control. The treatment with the hydrogels and saline was provided regularly, the wound size was measured on the 0th, 4th, 8th, 16th, 21st, and 28th days with a measuring scale, and photographs were taken specifically. The scar-free wound-healing process was observed and depicted in a figure.

#### 4.16.2. Histopathology

Histological analysis was crucial for understanding the quality of tissue regeneration promoted by the healing activity of the hydrogels. On the 28th day, the hair was removed, and the animals were euthanized by providing an overdose of Isoflurane. Later, tissue from all groups and normal skin from any part of the skin other than the wound area were also excised and indicated as the non-saline-treated (NST) group. The excised tissues were washed thrice with PBS and stored in 10% Formalin solution. Later, the tissues were embedded in paraffin, sliced into a thickness of 5 µm, placed on the microscopic slide, and stained with hematoxylin and eosin (H&E). Further, an optical microscope (Olympus CH20i) was utilized to visualize the tissue layers and the changes that occurred in the tissue with 10× and 40× magnification. The photographs, captured at different places and indicating the changes, were represented as a figure.

#### 4.16.3. Assessment of Organ and Body Weight

Similarly, to examine whether any toxicological changes had occurred, the organs were collected after euthanization. Organs like the heart, intestine, kidney, liver, spleen, and brain were collected and washed thrice with PBS. The corresponding weight was recorded, and the organs were stored in 10% formalin solution. The body weight was also recorded throughout the study, where the mean ± standard deviation was calculated and represented in a table. The relative organ weight was calculated as follows,

$$Relative\;organ\;weight\;\%=\frac{Organ\;weight}{Body\;weight\;of\;the\;animal\;on\;28th\;day}\times 100$$

#### 4.16.4. Biochemical Parameters

As oxygen and nutrient supply are critical parameters in wound healing, analyzing these changes provides valuable insights into the healing process and helps identify strategies to promote faster healing. To perform these, on the 7th and 28th days, about 2 mL of blood was collected from the retro-orbital plexus. Parameters such as hemoglobin, RBC, WBC, PCV, and platelet count and immune cells such as neutrophils, basophils, lymphocytes, monocytes, and eosinophils were analyzed. The analyzed parameters, along with statistical analysis, were represented in the form of a table.

#### 4.16.5. Growth Factor Analysis

Growth factors are essential key regulators of the wound healing process that deliver mechanistic proof of how hydrogel treatment promotes tissue repair and complete wound healing. Following tissue collection, growth factors, such as vascular endothelial growth factor (VEGF) and platelet-derived growth factor (PDGF), were analyzed to further confirm the changes observed in wound healing and histological data. The growth factor analysis was performed following the kit protocol for the tissues excised from all 6 groups. The kits, such as the Rat Platelet Derived Growth Factor (PDGF) ELISA Kit and the Elabscience Rat VEGF-A (Vascular Endothelial Cell) ELISA Kit, were utilized for the analysis. The analyzed changes were represented as a graphical image.

#### 4.16.6. Hydroxyproline Assay

Complete wound healing analysis requires a major assessment in estimating the level of collagen deposition that critically reflects the obtained results. Collagen deposition can be assessed by a biochemical marker called the hydroxyproline assay, which provides the level of deposited collagen to form the ECM. The hydroxyproline assay was carried out on the excised tissues from the following groups: NST, ST, AG-AgNP, and AG-AgNP-PE. The assay was performed following the kit’s protocol, and the levels of deposited collagen were analyzed and represented in a graphical form.

### 4.17. Statistical Analysis

The data are expressed as mean ± standard deviation (SD). The statistical analysis was performed using Microsoft Excel, Student’s *t*-test and one-way ANOVA. Differences were considered statistically significant at *p* < 0.05.

## Figures and Tables

**Table 1:** Characteristic FTIR peaks, along with the corresponding chemical bonds and functional groups, for all the developed hydrogels.

Mark	Wavenumber (cm^−1^)	Chemical Bond	Functional Groups
A	3398	O-H and N-H stretching, intermolecular H-bonding	Indicating O-H and N-H stretching vibrations in the polymeric matrix and intermolecular hydrogen bonding network
	1629	C=N/C=H stretching	Indicating crosslinking and chemical conjugation
B	2926	Asymmetric C-H stretch, O-H stretch	Presence of aliphatic chains
	2838	Symmetric C-H or methoxy stretching	Presence of polyphenols
	1509	C-C stretch (in ring)	Presence of Aromatics
	1034	C-N stretch, C-O stretch	Presence of Aromatic amines, alcohols, carboxylic acids, ethers, and esters
	955	Out-of-plane C-H bending/C-O stretching	Presence of glycosidic bonds and indication for crosslinking

**Table 2:** Percentage of wound closure accelerated by the hydrogel formulations in 72 h.

Hydrogel Formulation	Percentage of Wound Closure at 72 h (%)	
	0.5 µg/mL	5 µg/mL
Control	86%	
AG-AgNP	71%	65%
AG-AgNP-PE	96%	99%
AG-AgNP-FL	63%	71%
AG-AgNP-PL	78%	79%
AG-AgNP-AL	70%	81%
AG-AgNP-CU	67%	73%

**Table 3:** Histological changes observed in the excised healed tissues.

Group	Blue Arrow	Yellow Arrow	Red Arrow	Black Arrow	White Arrow
G1	Mild abnormal regeneration	Abnormal dermis	Increased fibroblast cells	Abnormal inflammatory cell infiltration	–
G2	Normal regeneration	Normal dermis	Granulation tissue with increased fibroblast cells	Increased inflammatory cells	–
G3	Normal regeneration	Mildly abnormal dermis	Granulation tissue with increased fibroblast cells	Disturbed inflammatory cell deposition	Disrupted healthy cells
G4	Normal regeneration	Uniform dermis	Mature granulation tissue with fewer fibroblast cells	Scattered inflammatory cells with thin capillary blood vessels	Normal healthy tissue with collagen bundles
G5	Normal regeneration	Uniform dermis	Mature granulation tissue with minimal fibroblast cells	Scattered inflammatory cells with thin capillary blood vessels	Normal healthy tissue layer
G6	Normal regeneration	Uniform dermis with normal compact collagen bundles	–	–	Healthy tissue

**Table 4:** Hematological analysis demonstrating the effect of the hydrogels and the control on day 7. The results are expressed as mean ± SD.

Parameter	Group 1	Group 2	Group 3	Group 4	Group 5
Hemoglobin	10.1 ± 0.505	10.6 ± 0.53	11.9 ± 0.595	9.6 ± 0.48	9.1 ± 0.455
RBC (10^12^/L)	11.0 ± 0.55	9.3 ± 0.465	8.8 ± 0.44	7.3 ± 0.365	10.3 ± 0.515
WBC (10^9^/L)	6.4 ± 0.32	8.1 ± 0.405	11.0 ± 0.55	6.0 ± 0.30	8.9 ± 0.445
Platelet Count (10^9^/L)	9000 ± 450	9500 ± 475	7200 ± 360	5200 ± 260	7300 ± 365
PCV (%)	45.7 ± 2.285	39.8 ± 1.99	40.6 ± 2.03	48.2 ± 2.41	39.9 ± 1.995
MCV (fL)	55.2 ± 2.76	50.4 ± 2.52	45.2 ± 2.26	33.6 ± 1.68	40.6 ± 2.03
MCH (pg)	9.95 ± 0.4975	9.1 ± 0.455	10.5 ± 0.525	10.5 ± 0.525	9.8 ± 0.49
MCHC (g/dL)	20.4 ± 1.02	26.1 ± 1.305	36.1 ± 1.805	31.8 ± 1.59	33.0 ± 1.65
MPV (fL)	4.6 ± 0.23	5.0 ± 0.25	5.3 ± 0.265	4.8 ± 0.24	7.3 ± 0.365
Neutrophils (%)	28 ± 1.4	24 ± 1.2	20 ± 1.0	31 ± 1.55	16 ± 0.8
Lymphocytes (%)	66 ± 3.3	73 ± 3.65	78 ± 3.9	65 ± 3.25	81 ± 4.05
Monocytes (%)	5 ± 0.25	2 ± 0.10	1 ± 0.05	3 ± 0.15	2 ± 0.10
Eosinophils (%)	1 ± 0.05	1 ± 0.05	1 ± 0.05	1 ± 0.05	1 ± 0.05
Basophil (%)	0 ± 0	0 ± 0	0 ± 0	0 ± 0	0 ± 0

**Table 5:** Hematological analysis demonstrating the effect of the hydrogels and the control on day 28. The results are expressed as mean ± SD.

Parameter	Group 1	Group 2	Group 3	Group 4	Group 5
Hemoglobin	9.6 ± 0.48	12.8 ± 0.64	12.2 ± 0.61	13.02 ± 0.651	12.5 ± 0.625
RBC (10^12^/L)	8.7 ± 0.435	9.0 ± 0.45	9.8 ± 0.49	7.2 ± 0.36	9.6 ± 0.48
WBC (10^9^/L)	9.6 ± 0.48	13.6 ± 0.68	6.6 ± 0.33	11.0 ± 0.55	8.4 ± 0.42
Platelet Count (10^9^/L)	688 ± 34.4	1268 ± 63.4	1035 ± 51.75	1470 ± 73.5	1130 ± 56.5
PCV (%)	40.5 ± 2.025	43.8 ± 2.19	40.8 ± 2.04	48.7 ± 2.435	38.9 ± 1.945
MCV (fL)	58.7 ± 2.935	49.0 ± 2.45	46.3 ± 2.315	42.0 ± 2.1	40.2 ± 2.01
MCH (pg)	11.1 ± 0.555	14.3 ± 0.715	15.1 ± 0.755	12.6 ± 0.63	11.7 ± 0.585
MCHC (g/dL)	23.6 ± 1.18	29.1 ± 1.455	31.2 ± 1.56	31.8 ± 1.59	28.6 ± 1.43
MPV (fL)	5.0 ± 0.25	4.8 ± 0.24	5.8 ± 0.29	4.8 ± 0.24	3.3 ± 0.165
Neutrophils (%)	28 ± 1.4	18 ± 0.9	29 ± 1.45	15 ± 0.75	19 ± 0.95
Lymphocytes (%)	67 ± 3.35	79 ± 3.95	68 ± 3.4	80 ± 4.0	76 ± 3.8
Monocytes (%)	3 ± 0.15	2 ± 0.1	2 ± 0.1	1 ± 0.05	3 ± 0.15
Eosinophils (%)	2 ± 0.1	1 ± 0.05	1 ± 0.05	2 ± 0.1	2 ± 0.1
Basophils (%)	0 ± 0	0 ± 0	0 ± 0	0 ± 0	0 ± 0

**Table 6:** Distribution of groups and concentrations used for the CAM assay.

**Groups**	**Concentration**	**No. of Eggs**
Positive control (Heparin treated)	100 µL of 5000 IU/mL stock	5
Negative control (Untreated)	-	5
AG-AgNP (High)	5 µg/mL	5
AG-AgNP (Low)	2.5 µg/mL	5
AG-AgNP-PE (High)	5 µg/mL	5
AG-AgNP-PE (Low)	2.5 µg/mL	5

## Data Availability

The data will be made available by the corresponding author on reasonable request.

## Supplementary Materials

The following supporting information can be downloaded at: https://www.mdpi.com/article/10.3390/gels12080719/s1, Table S1: The relative body weight measured for the experimental animals during the study period. The values are expressed as mean ± SD. Table S2: Relative organ weight of the experimental animals measured during the study period. The values are expressed as mean ± SD.

## Abbreviations

AG-AgNP	Alginate + gelatin + silver nanoparticle hydrogel
AG-AgNP-PE	Alginate + gelatin + silver nanoparticle + combined plant extracts
AG-AgNP-FL	Alginate + gelatin + silver nanoparticle + flower extract
AG-AgNP-PL	Alginate + gelatin + silver nanoparticle + plantain peel extract
AG-AgNP-AL	Alginate + gelatin + silver nanoparticle + aloe vera extract
AG-AgNP-CU	Alginate + gelatin + silver nanoparticle + curcumin
AgNPs	Silver nanoparticles
CAM	Chorioallantoic membrane
DMSO	Dimethyl sulfoxide
DPPH	2,2-Diphenyl-1-picrylhydrazyl
ECM	Extracellular matrix
*E. coli*	*Escherichia coli*
ELISA	Enzyme-linked immunosorbent assay
FTIR	Fourier-transform infrared spectroscopy
H&E	Hematoxylin and eosin staining
IAEC	Institutional Animal Ethics Committee
MIC	Minimum inhibitory concentration
MTT	3-(4,5-Dimethylthiazol-2-yl)-2,5-diphenyltetrazolium bromide
NST	Non-saline-treated
OD	Optical density
PBS	Phosphate-buffered saline
PCV	Packed cell volume
PDGF	Platelet-derived growth factor
RBC	Red blood cells
ROS	Reactive oxygen species
*S. aureus*	*Staphylococcus aureus*
SSD	Silver sulfadiazine
ST	Saline-treated
TGF-β1	Transforming growth factor beta 1
UV	Ultraviolet
VEGF	Vascular endothelial growth factor
V79	Chinese hamster lung fibroblast cell line
WBC	White blood cells
