# Supplementary material for: Multifunctional Hydrogel with Phytochemicals and Silver Nanoparticles for Promoting Scar-Free Wound Healing
Source: Gels. 2026 Aug 13;12(8):719. doi: 10.3390/gels12080719 (PMC13512311; doi:10.3390/gels12080719)
Supplement: Supplementary file 1 [file gels-12-00719-s001.zip › gels-4456542-supplementary.pdf]

Table S1: The relative body weight measured for the experimental animals during the study period. The values are expressed as mean ± SD.

Group	Day 0	Day 4	Day 8	Day 16	Day 21
G1	157.23 ± 3.46 g	166.07 ± 2.83 g	183.07 ± 5.78 g	221.93 ± 10.46 g	261.13 ± 9.30 g
G2	153.33 ± 17.97g	165.13 ± 20.39 g	184.00 ± 24.99 g	224.47 ± 22.28 g	262.40 ± 18.57 g
G3	189.40 ± 6.68 g	194.27 ± 11.77 g	205.53 ± 13.61 g	238.80 ± 11.85 g	277.67 ± 11.04 g
G4	208.73 ± 54.08g	218.07 ± 56.14 g	234.67 ± 61.70 g	268.20 ± 64.07 g	303.33 ± 51.74 g
G5	160.13 ± 11.04g	169.33 ± 8.41 g	178.53 ± 15.51 g	225.60 ± 5.03 g	263.27 ± 13.57 g

Table S2: Relative organ weight of the experimental animals measured during the study period. The values are expressed as mean ± SD.

Parameter	Group 1	Group 2	Group 3	Group 4	Group 5
**Heart (%)**	0.36 ± 0.03	0.40 ± 0.06	0.37 ± 0.04	0.34 ± 0.02	0.43 ± 0.08
**Intestine (%)**	0.27 ± 0.20	0.27 ± 0.17	0.23 ± 0.21	0.27 ± 0.11	0.27 ± 0.13
**Kidney (%)**	0.39 ± 0.14	0.44 ± 0.15	0.45 ± 0.29	0.36 ± 0.02	0.48 ± 0.24
**Liver (%)**	4.23 ± 1.19	3.33 ± 0.08	4.55 ± 0.29	3.22 ± 1.39	4.06 ± 1.61
**Spleen (%)**	0.29 ± 0.09	0.29 ± 0.03	0.27 ± 2.36	0.19 ± 0.06	0.46 ± 0.61
**Brain (%)**	0.58 ± 0.06	0.63 ± 0.04	0.57 ± 0.07	0.51 ± 0.06	0.60 ± 0.01
